# Does pubertal stage mediate the association between family environment and structure and function of the amygdala-mPFC circuit? A replication study of the longitudinal ABCD cohort

**DOI:** 10.1016/j.dcn.2022.101120

**Published:** 2022-06-10

**Authors:** Sandra Thijssen, Paul F. Collins, Monica Luciana

**Affiliations:** a Behavioral Science Institute, Radboud University, Nijmegen, the Netherlands; b Department of Psychology, Education, and Child Studies, Erasmus University Rotterdam, the Netherlands; c Department of Psychology, University of Minnesota, Minneapolis, MN, USA

**Keywords:** amygdala-mPFC, amygdala, family environment, accelerated development, pubertal development

## Abstract

Psychosocial acceleration theory suggests that early stress accelerates pubertal development. Using half of the baseline Adolescent Brain and Cognitive Development (ABCD) cohort, Thijssen et al. (2020) provide support that accelerated puberty following stressful family environments may promote neurodevelopment. Here, we replicate and extend those analyses using 1) data from the second half of the ABCD sample (n = 3300 +, ages 9–10), and 2) longitudinal imaging data from the original sample (n = 1800 +, ages 11–12). A family environment latent variable was created and related to anterior cingulate cortex (ACC) thickness, area, white matter fractional anisotropy, amygdala volume, and cingulo-opercular network (CON)–amygdala resting-state functional connectivity. Results from the independent sample replicate the mediating effects of family environment through pubertal stage on amygdala-CON functional connectivity. Sex-stratified analyses show indirect effects via pubertal stage in girls; boys show evidence for direct associations. Analyses using wave 2 imaging data or wave 2-wave 1 difference scores from the originally-analyzed sample replicate the resting-state indirect effects. The current paper replicates the mediating role for puberty in the association between family environment and neurodevelopment. As both direct and indirect associations were found, puberty may be one of multiple mechanisms driving accelerated neurodevelopment following environmental stress.

As the primary source of protection and comfort, parental care is an important predictor of child development. Even as the child develops independence, parental care and the larger familial context continue to exert substantial influences on child development. While factors like parental warmth have been associated with increased child well-being (Rothenberg et al., 2020), negative factors like parent-child conflict, harsh discipline or parental psychopathology are linked with increased internalizing and externalizing problems (e.g. Weijers et al., 2018; Xerxa et al., 2021), and deviations in neural and physical development (e.g. Cortes Hidalgo et al., 2021; Ellis and Garber, 2000). Historically, such deviations in response to stressful family factors have been interpreted as impairments, but more recently, they are viewed as adaptations to the stressful environment (Ellis et al., 2017). For example, evolutionary life history theories, such as the psychosocial acceleration theory, suggest that early familial stress accelerates pubertal development to increase reproductive fitness (Belsky et al., 1991). Relatedly, recent research suggests that development of the amygdala-medial prefrontal cortex (mPFC) circuit, which is involved in emotion regulation, is accelerated in response to early stressful family circumstances (Gee et al., 2013a, Herzberg et al., 2021, Thijssen et al., 2017). Early maturation of this circuit allows the child to self-regulate emotions instead of relying on parents who are unable to provide the proper care (Callaghan and Tottenham, 2016). The current paper aims to replicate and extend results by Thijssen et al. (2020) who sought to bridge these two findings by examining if the association between a stressful family environment and structure and function of the amygdala-mPFC circuit is mediated by accelerated pubertal development, using baseline data from approximately half of the participants in the Adolescent Brain Cognitive Development (ABCD) study.

The amygdala, a subcortical temporal lobe structure, plays a role in emotional learning and facilitates attention to salient cues (Phelps and LeDoux, 2005). The mPFC, including the anterior cingulate cortex (ACC), is implicated in emotional and cognitive functioning (Forbes and Grafman, 2010, Ridderinkhof et al., 2004) and may provide top-down regulation of amygdala reactivity to emotional stimuli. Consequently, functional coupling of these two regions may be involved in emotion regulation (Hariri et al., 2003, Ochsner et al., 2002, Phan et al., 2005). In childhood, parents play an important role in child emotion regulation (Tottenham, 2015), but as the amygdala-mPFC circuit matures, children become increasingly capable of self-regulation. This transition from parent-guided to self-regulation is nicely illustrated by fMRI studies on fear processing. In children, responses of the amygdala and mPFC to fearful faces are positively correlated (Gee et al., 2013b), whereas adolescents and adults show negative patterns of amygdala-mPFC functional connectivity in response to the same stimuli. This negative pattern of functional connectivity is interpreted as effective top-down control of the amygdala by the mPFC (however, using a multiverse approach Bloom et al., 2021 did not find evidence for age related changes in amygdala-mPFC functional connectivity in response to this task).

Recent studies suggest that the development of the amygdala-mPFC circuit is accelerated in children experiencing early life family stress (Gee et al., 2013a, Herringa et al., 2016, Herzberg et al., 2021, Thijssen et al., 2020, Thijssen et al., 2017). Gee, Gabard-Durnam et al. (2013a) showed that previously institutionalized youth may demonstrate negative amygdala-mPFC connectivity in response to fearful faces earlier in development than family-reared youth, suggesting accelerated development in response to extreme early life adversity. Importantly, stress can be considered from a dimensional perspective. Evidence for accelerated development has been reported in response to normative variations in the family environment (e.g., low levels of sensitive parental care, Thijssen et al., 2017; Thijssen et al., 2020). It is, however, unclear what mechanisms explain how stressful environments accelerate neural development. Possibly, the release of cortisol in response to early stress directly affects amygdala–mPFC circuit development (Callaghan and Tottenham, 2016; Gee et al., 2013a). Alternatively, pubertal hormones may play a role.

A large literature suggests that early stress is associated with accelerated pubertal development. This acceleration is predicted by the psychosocial acceleration theory (Belsky et al., 1991), which suggests that children adjust their developmental trajectories to match their environment. The psychosocial acceleration theory proposes that parental care and investment provide children with information about availability and predictability of resources and relationships, with less than optimal care suggesting scarcity of resources and interpersonal relationship quality. In such an environment, it may be more beneficial to take a quantitative rather than qualitative approach to reproduction. Thus, early pubertal timing may increase reproductive fitness. Indeed, several studies have shown that factors ranging from adversities such as abuse (Mendle et al., 2016) to more normative experiences of family related stress like harsh discipline (Belsky et al., 2010), father absence (Aghaee et al., 2020), or parental psychopathology (Ellis and Essex, 2007, Ellis and Garber, 2000) are related to accelerated pubertal development, while higher quality parental investment and support relate to slower pubertal development (Ellis and Essex, 2007).

Pubertal hormones have organizing effects on the brain (Goddings et al., 2014, Herting et al., 2015, Piekarski et al., 2017). Accelerated pubertal development may therefore shape subsequent neurodevelopment. In female rodents, estradiol has been shown to increase inhibitory ACC activation possibly tipping the excitatory/inhibitory balance regulating ACC sensitive-period plasticity (Piekarski et al., 2017). Piekarski et al. (2017) showed that pre-pubertal, but not post-pubertal gonadectomy blocked this increase in inhibitory activation and that hormone administrations in pre-pubertal gonadectomized animals restore this increase in inhibitory activation.

Evidence for accelerated neurodevelopment following early pubertal timing in human studies is mixed. Dehydroepiandrosterone, an adrenarcheal hormone, has been related to increased (or decreased negative) amygdala-prefrontal connectivity during emotion processing (Barendse et al., 2020a), suggesting decelerated rather than accelerated neurodevelopment. Studies examining other modalities of neural development provide conflicting results, with some studies suggesting minimal effects of puberty (Barendse et al., 2020b), while others suggest that accelerated pubertal development could accelerate (Barendse et al., 2018, Vijayakumar et al., 2021a, Wierenga et al., 2018) or decelerate neurodevelopment (Klauser et al., 2015).

Using data from the ABCD Study, Thijssen et al. (2020) cross-sectionally examined whether associations between the child’s family environment and the amygdala-mPFC circuit were mediated by pubertal stage. While most evidence has focused on accelerated functional development of the amygdala–mPFC circuit (Gee et al., 2013a, Thijssen et al., 2017), structural development may also be impacted. Through an analysis of the ABCD study’s structural and intrinsic functional connectivity data, Thijssen et al. (2020)’s results suggested that a more stressful family environment relates to both function and structure of the amygdala-mPFC circuit directly, but also through associations with a more advanced pubertal stage. Specifically, family environment was associated with decreased amygdala-cingulo-opercular network functional (used proxy for amygdala-mPFC functional connectivity), increased ACC fractional anisotropy (FA) and decreased ACC cortical thickness via pubertal stage. As FA increases and thickness decreases over adolescence (Lebel and Deoni, 2018, Vijayakumar et al., 2016, Walhovd et al., 2016), and as Brieant et al. (2021) show that amygdala-CON functional connectivity decreased between wave 1 and wave 2 of the ABCD study, these findings suggest that accelerated pubertal development in response to early family stress may accelerate development of the amygdala-mPFC circuit. Replication of these initial results would further substantiate that accelerated pubertal development may affect and possibly accelerate neural development. Moreover, an important limitation of Thijssen et al. (2020) is the cross-sectional nature of the analysis. Therefore, the aim of the current paper is to replicate the results by Thijssen et al. (2020) and to extend these findings using longitudinal data.

In the original study, Thijssen et al. used data from the first data release of the ABCD Study, which has enrolled a total of 11,878 participants. This first data release included data on only half of the baseline sample (n = 4524). In 2019, the remaining baseline data were released. This biphasic release of the baseline data creates the opportunity to replicate the analyses performed in Thijssen et al. (2020) using identical measures and procedures. In 2020, ABCD made available new imaging data collected approximately 2 years after the baseline session for the first half of the intended sample, allowing a longitudinal analyses of the original sample. The current study replicates and extends Thijssen et al. (2020)’s analyses using 1) cross-sectional analysis of the baseline data of the remaining half of the ABCD sample, and 2) longitudinal wave 2 imaging data as well as wave 2 - wave 1 imaging difference scores from the original Thijssen et al. (2020) sample. We hypothesize that in both the cross-sectional and longitudinal analyses, associations between a more stressful family environment and structure and function of the amygdala-mPFC circuit are mediated by accelerated pubertal development (i.e. decreased thickness in wave 1 and wave 2/greater thinning between wave 1 and wave 2; increased FA in wave 1 and wave 2/greater increase in FA between wave 1 and wave 2; decreased functional connectivity in wave 1 and wave 2/greater decrease in functional connectivity between wave 1 and wave 2).

## 1 Methods

### 1.1 Participants

The present study used data collected for the ABCD Study (http://dx.doi.org/10.15154/1412097, http://dx.doi.org/10.15154/1503209, and http://dx.doi.org/10.15154/1519007). The ABCD Study is following a population-based, prospective cohort of 11.875 children from ages 9–10 years into adulthood. Data are collected across 21 United States sites. ABCD’s recruitment strategies have been described elsewhere (Garavan et al. (2018). Parents provided informed consent, and children provided assent to participate. Data collection was approved by a centralized internal review board of the University of San Diego, as well as by the review boards of all research sites.

#### 1.1.1 Study 1: cross-sectional replication

The replication study utilized data primarily derived from ABCD data release 3.0 with one exception as indicated below. Exclusion criteria were identical to Thijssen et al. (2020). Of the 11,878 children, we excluded 561 participants who attended their research sessions supervised by someone other than their biological parent, in order to increase the validity of the parent reported measures. 335 children were excluded due to MRI incidental findings. 2075 twins were excluded, and from each of 367 sibling pairs, one child was randomly excluded from the analysis. From an additional 337 sibling pairs, one sibling had unusable imaging data and was excluded (see Quality Assurance). Finally, all children who were part of the Thijssen et al. (2020) sample were excluded from the cross-sectional replication sample (n = 3001). These exclusions yielded a final sample of 5199 children, of whom 4316 participants had good quality MRI T1 data, 3892 had good quality rsfMRI data, and 3360 had good quality DTI data (see Quality Assurance). Fig. S1 provides a flowchart of the exclusion procedures.

#### 1.1.2 Study 2: longitudinal analysis

The longitudinal analyses included data from ABCD releases 1.0 (family environment data and pubertal development data) and 3.0 (imaging data). From the original MRI T1 sample of 2495 children, 2016 children had available T1 MRI data at wave 2, of which 1878 children had good quality T1 data. For the rsfMRI analyses, 1977 of the original 2461 participants had available rsfMRI data at wave 2, of which 1925 had good quality data. Fig. S1 presents a flowchart of the exclusion procedures for study 2.

### 1.2 Measures

#### 1.2.1 Family environment

Prior literature suggests that different experiences related to the family environment, ranging from socioeconomic status (Sun et al., 2017) to father absence (Aghaee et al., 2020) and parenting quality (Belsky et al., 2010, Ellis and Essex, 2007), are related to pubertal development. As these factors often do not occur in isolation, a latent factor was constructed reflecting the quality of family environment using Mplus (Muthén and Muthén, 2007). Three types of information were used: child-reported information about family dynamics and relationships, parent-reported information about family dynamics and relationships, and demographic information (Thijssen et al., 2020). Variables related to these three topics were first combined into child, parent and demographics latent variables. In the same model, these latent variables were combined to yield an overall family environment variable. For detailed information on questionnaires used and modelling, see Supplemental Text 1. To improve model fit, parental psychopathology, which was originally modelled as part of the demographic score, was now modelled as part of the parental score. The Family Environment score was not significantly affected by this change (correlation between scores *r =* 0.988).

Low scores on the family environment variable indicate a more stressful/less supportive environment (i.e., increased family conflict, lower parental acceptance and monitoring, lower socioeconomic status, and/or higher parental psychopathology). See Table S1 for correlations between the latent variables.

#### 1.2.2 Pubertal stage

Parents reported on the child’s pubertal stage using the Pubertal Development Scale (Petersen et al., 1988). See Supplemental Tables S2a-d for the distributions of pubertal stage in each analyzed sub-sample. Due to low numbers of children in pubertal stages 4 and 5, we combined stages 3, 4, and 5 as Stage 3 + (as in Thijssen et al., 2020).

#### 1.2.3 Magnetic resonance imaging

##### 1.2.3.1 Measures of the amygdala–mPFC circuit

ABCD provides tabulated summary statistics of MRI data based on processing algorithms implemented by its data analytic core (Hagler et al., 2019). Given that both Gee et al. (2013a) and Thijssen et al. (2017) report correlations between familial environment and amygdala–ACC (which is part of the mPFC) connectivity, Thijssen et al. (2020) focused on indices of amygdala and ACC gray and white matter structure. As such, we examined amygdala volumes, ACC cortical thickness and surface area, and ACC white matter fractional anisotropy. Unfortunately, no direct measure of amygdala–mPFC functional connectivity had been released by ABCD at time of the original study, but amygdala functional connectivity with several well-characterized resting-state networks (Gordon et al., 2016) was provided in the first data release. Therefore, associations between the amygdala and the cingulo-operculum network, which includes the caudal ACC and insula, were assessed.

##### 1.2.3.2 MRI acquisition and preprocessing

For details on MRI acquisition and data preprocessing in the ABCD Study, see Casey et al. (2018) and Hagler et al. (2019). Supplemental text 2 includes information on the sequences used and preprocessing for the current study as well as information on quality assurance.

The DTI data released in ABCD’s third release (3.0) were preprocessed using a different preprocessing stream as compared to the first data release. Because of this change in ABCD’s processing stream, it is not possible to fully assess the DTI data replicability of our model. Therefore, in Study 2 (longitudinal analysis) we did not perform analyses using DTI data. For Study 1, DTI data from the second data release (2.0.1) were used, because these data represent the full baseline sample and were processed by ABCD using similar methods as the 1.0 release (which was the basis of the Thijssen et al., 2020 report).

### 1.3 Statistical approach

To test the hypothesized indirect effect of family environment on structure and function of the amygdala–mPFC circuit via pubertal stage, linear mediation analyses were performed in Mplus, correcting for child sex assigned at birth, age, and race. For study 1, family environment, pubertal stage and the neuroimaging data were measured at wave 1. For study 2, family environment and pubertal stage were measured at wave 1, whereas structure and function of the amygdala-mPFC circuit was measured at wave 2. We additionally examined whether family environment and pubertal stage (measured at wave 1) were associated with neural development from wave 1 to wave 2 by examining MRI wave 2-wave 1 difference scores as outcomes (correcting for MRI score at wave 1). We also calculated pubertal change scores from wave 1 to wave 2, but because the family environment measure did not predict pubertal change from wave 1 to wave 2 (correcting for pubertal stage at wave 1, age, sex and ethnicity), we did not assess mediation models (girls, β = 0.08, *p* = .177, and boys, β = 0.07, *p* = .175). Because relevant theories and prior literature suggest that early environment may accelerate development, we did not examine change scores in family environment from wave 1 to wave 2.

Brain measures were residualized for study site (dummy coded), and whole-brain volume (gray matter measures). Due to the large variation of the cortical surface area and subcortical volume measures, these measures were converted to z-scores. Separate models were run for each modality of the same structure (MRI T1 ACC, MRI T1 amygdala, DTI, and resting-state fMRI). Models were initially run on the full sample. To assess sex differences, we performed mediation analyses stratified by sex. For all analyses, α = 0.05 (2-sided). P-values < 0.10 were interpreted as trends. P-values were FDR-corrected for multiple testing per structure within sample (total sample, boys, girls) (Benjamini and Hochberg, 1995).

Because gray matter development may follow an inverted U-shaped developmental trajectory with peaks of gray matter in early adolescence (Giedd et al., 1999, Mills et al., 2016, Vijayakumar et al., 2016), the distribution of the gray matter metrics across pubertal stages was visualized (see Fig. S4 and S5). For study 2, ACC cortical surface area at wave 2 and ACC cortical thickness and surface area change scores showed evidence of a quadratic association with pubertal stage. For these outcomes, quadratic mediation was also tested (quadratic association between mediator pubertal stage and gray matter outcome) using the Medcurve macro in SPSS (Hayes and Preacher, 2010). However, no evidence for nonlinear mediation was found. Finally, because the family environment variable is meant to capture normative variation in family-related stress, we performed sensitivity analyses controlling for traumatic events using the parent-reported Post-Traumatic Stress section from the Kiddie Schedule for Affective Disorders and Schizophrenia in the total sample.

## 2 Results

### 2.1 Study 1: cross-sectional replication study

Tables S3a and S3b provide characteristics of the sample used to create the Family Environment score (i.e. including individuals with poor quality MRI data). As expected, there were significant differences in pubertal development between the sexes, with more girls in higher stages compared to boys (χ^2^ (2) = 808.19, *p* < .001). Boys (*M* = −0.11, *SD* = 0.59) had a lower FE score (e.g., lower quality environment) compared to girls (*M* = 0.01, *SD* = 0.60), *t*(5195) = 6.788, *p* < .001.

Correlations between the outcomes of interest as well as sex-corrected correlations with age can be found in Table S4. Associations between family environment and menarche are provided in Supplemental Text 3.

#### 2.1.1 Study 1 mediation analyses

**Fig. 1:**
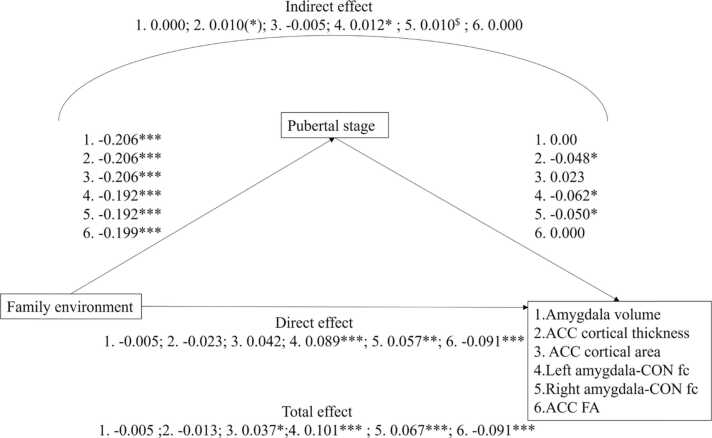
Mediation models Study 1. Values are standardized coefficients. ^$^*p* < .10, (*) *p*_uncorrected_ < 0.05, * *p* < .01, ** *p* < .01, *** *p* < .001. ACC = anterior cingulate cortex; CON = cingulu-opercular network; fc = functional connectivity; FA = fractional anisotropy.

For a visual representation of the mediation models, see Fig. 1.

##### 2.1.1.1 Structural MRI

For full model coefficients, see Tables S6-S8, for total sample, girls and boys respectively.

###### 2.1.1.1.1 Amygdala volume

Similar to Thijssen et al. (2020), for amygdalae volume, the total, direct (effect controlled for pubertal stage), and indirect (effect via pubertal stage) effects of family environment were not significant: β = −0.005, *p*_corr_ = 0.868, β = −0.005, *p*_corr_ = 0.994, β = 0.000, *p*_corr_ = 0.994, for total, direct, and indirect effects, respectively.

Results for the sex-stratified analyses can be found in Table S5. None of the associations were significant.

###### 2.1.1.1.2 Cortical thickness

Although the effect did not survive correction for multiple testing, similar to the original study, only the indirect effect of family environment on ACC cortical thickness showed evidence of an effect: β = −0.013, *p*_corr_ = 0.863, β = –0.023, *p*_corr_ = 0.492, β = 0.010, *p*_corr_ = 0.108 (*p* = .043), for total, direct and indirect effects, respectively.

For results of the sex stratified analyses, see Table S5. Contrary to the 2020 paper, we did not find significant indirect effects in girls.

###### 2.1.1.1.3 Cortical surface area

Unlike the original study, where no linear associations were found for cortical surface area, here, we report a significant total and direct effect of family environment on ACC cortical surface area, β = 0.037, *p*_corr_ = 0.048, β = 0.042, *p*_corr_ = 0.024, for total and direct effects, respectively. The indirect effect of family environmental on ACC cortical surface area through pubertal stage was not significant, β = −0.005, *p*_corr_ = 0.756.

For the sex stratified analyses, see Table S5. In girls, significant total and direct effects were found, β = 0.062, *p*_corr_ = 0.012, β = 0.060, *p*_corr_ = 0.024 for total and direct effects, respectively.

##### 2.1.1.2 Resting-state fMRI

For full model coefficients, see Table S9-S11. In the original study, significant total and direct effects were found for amygdala-cingulo-opercular network functional connectivity. For both left and right amygdala functional connectivity, the indirect effects indicated a trend in the expected direction. In the replication sample, the total, direct, and indirect effects of family environment on cingulo-opercular network–left amygdala functional connectivity were significant, β = 0.101, *p*_corr_ < 0.001, β = 0.089, *p*_corr_ < 0.001, β = 0.012, *p*_corr_ = 0.028, respectively. For cingulo-opercular network– right amygdala functional connectivity, the total, direct, and indirect effects were β = 0.067, *p*_corr_ < 0.001, β = 0.057, *p*_corr_ = 0.003, β = 0.010, *p*_corr_ = 0.058, respectively. Thus, a lower quality family environment was related to weaker functional connectivity between the cingulo-opercular network and the amygdalae directly, but also through its association with a more advanced pubertal stage (for right amygdala, there is a trend in expected direction only).

For results of the sex stratified analyses, see Table S5. Similar to the original study, the indirect effects are more prominent for girls. In girls, the total, direct, and indirect effects of family environment on cingulo-opercular network–left amygdala functional connectivity were significant: β = 0.108, *p*_co__r__r_ < 0.001, β = 0.090, *p*_corr_ < 0.001, β = 0.018, *p*_corr_ = 0.028. For cingulo-opercular network– right amygdala functional connectivity, the total, direct, and indirect effects were β = 0.061, *p*_corr_ = 0.018, β = 0.048, *p*_corr_ = 0.111, β = 0.013, *p*_corr_ = 0.090, respectively. In boys, only the total and direct effects of family environment on cingulo-opercular network–amygdala functional connectivity were significant, β = 0.092, *p*_corr_ < 0.001, β = 0.088, *p*_corr_ < 0.001, for left amygdala functional connectivity, and β = 0.071, *p*_corr_ = 0.004, β = 0.067, *p*_corr_ = 0.012, for right amygdala functional connectivity, respectively. Thus, whereas for girls, family environment was associated with cingulo-opercular functional connectivity both directly and through pubertal stage, for boys, only the direct association was significant.

##### 2.1.1.3 White matter integrity: fractional anisotropy

For full model coefficients, see Table S12-S14. For ACC fractional anisotropy, the 2020 study reported significant total, direct, and indirect effects. In the replication sample, only the total and direct effects were significant: β = −0.091, *p* < .001 (total), β = −0.091, *p* < .001 (direct), β = 0.000, *p* = .998 (indirect).

For results of the sex stratified analyses, see Table S5. The sex stratified analyses showed significant total and direct effects in both sexes: β = −0.098, *p* < .001, β = −0.097, *p* < .001, for girls, and β = −0.081, *p* = .001, β = −0.081, *p* = .002, for boys. Thus, a more stressful family environment was related to increased ACC FA, but not via accelerated pubertal development.

### 2.2 Study 2: longitudinal analysis

Sample characteristics for the longitudinal analysis can be found in Table S3a and S3b. For correlations between the latent variables, see Thijssen et al. (2020). Correlations between the outcomes of interest as well as correlations with age can be found in Table S14.

#### 2.2.1 Neurodevelopmental timing: wave 2 analyses

**Fig. 2:**
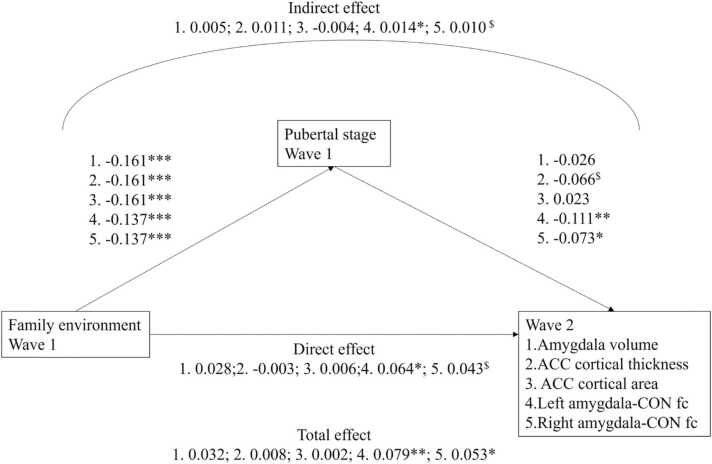
Mediation models Study 2. Values are standardized coefficients. ^$^*p* < .10, * *p* < .01, ** *p* < .01, *** *p* < .001. ACC = anterior cingulate cortex;; CON = cingulo-opercular network; fc = functional connectivity.

Wave 2 analyses examined family environment and pubertal stage at wave 1, but neural structure and functioning at wave 2. For a visual representation of the mediation models, see Fig. 2.

##### 2.2.1.1 Structural MRI

For full model coefficients, see Tables S17-S19, for total sample, girls and boys respectively.

###### 2.2.1.1.1 Amygdala volume

When examining the associations between wave 1 family environment, wave 1 pubertal stage and wave 2 amygdala volume, no significant associations were found: β = 0.032, *p*_corr_ = 0.438 (total), β = 0.028, *p*_corr_ = 0.708 (direct), β = 0.005, *p*_corr_ = 0.994 (indirect).

Results for the sex-stratified analyses can be found in Table S16. After correction for multiple comparisons, there were no significant effects.

###### 2.2.1.1.2 ACC cortical thickness

For ACC cortical thickness data at wave 2 in the original sample, no significant effects were found: β = 0.008, *p*_corr_ = 0.863 (total), β = −0.003, *p*_corr_ = 0.909 (direct), β = 0.011, *p*_corr_ = 0.108 (indirect).

Results for the sex-stratified analyses can be found in Table S16. In girls, a significant indirect effect of family environment on ACC cortical thickness was found, which did not survive correction for multiple testing, β = 0.020, *p*_corr_ = 0.138 (p = .046).

###### 2.2.1.1.3 ACC cortical surface area

For ACC cortical surface area, no significant effects were found: β = 0.002, *p*_corr_ = 0.909 (total), β = 0.006, *p*_corr_ = 0.798 (direct), β = −0.004, *p*_corr_ = 0.756 (indirect).

Results for the sex-stratified analyses can be found in Table S16. No significant associations were found.

##### 2.2.1.2 Resting-state fMRI

For full model coefficients, see Tables S20-S22. For left amygdala-cingulo-opercular functional connectivity, the total, direct, and indirect were β = 0.079, *p*_corr_ = 0.001, β = 0.064, *p*_corr_ = 0.016, β = 0.014, *p*_corr_ = 0.021, respectively. For right amygdala functional connectivity, the total, direct, indirect effect β = 0.053, *p*_corr_ = 0.020, β = 0.043, *p*_corr_ = 0.067, β = 0.010, *p*_corr_ = 0.058, respectively. A more stressful family environment was associated with lower amygdala-cingulo-opercular functional connectivity, both directly and indirectly via pubertal stage (for right amygdala, trend in expected direction only). The indirect effects were confirmed using a split-half replication, see Table S30. Although, due to the smaller sample size, not all effects are significant (e.g. p’s for indirect effect of left amygdala are.045 and.063), the effect sizes are of similar magnitude (e.g. β’s for the left amygdala indirect effect are 0.014 and 0.017 vs 0.014 in the total sample).

Results for the sex-stratified analyses can be found in Table S16. Stratified analyses suggest that the indirect effect was driven by girls (β = 0.023, *p*_corr_ =0.028, and β = 0.013, *p*_corr_ =0.149, for left and right amygdala functional connectivity, respectively), whereas the direct effect of family environment on amygdala-cingulo-opercular network functional connectivity was driven by boys (β = 0.087, *p*_corr_ =0.012, and β = 0.058, *p*_corr_ =0.052, for left and right amygdala functional connectivity, respectively).

#### 2.2.2 Neurodevelopmental tempo: wave 2 – wave 1 difference scores

There were no significant associations between the child’s family environment or pubertal status and wave 2- wave 1 differences scores for MRI T1w data. See Supplemental Text 4, and Tables S24-S29.

For the rs-fMRI data, significant total, direct, and indirect effects were found for left amygdala-cingulo-opercular network functional connectivity, β = 0.048, *p*_corr_ = 0.006, β = 0.038, *p*_corr_ = 0.042, β = 0.010, *p*_corr_ = 0.021. For right amygdala functional connectivity, the total effect was significant, whereas the indirect effect was not significant, but showed a trend in the expected direction, β = 0.032, *p*_corr_ = 0.048, β = 0.025, *p*_corr_ = 0.126, β = 0.007, *p*_corr_ = 0.058, for total, direct, and indirect effect, respectively. Thus, especially for left amygdala-cingulo-opercular network functional connectivity, a more stressful family environment was associated with a larger decrease in functional connectivity directly, but also via its association with a higher pubertal stage. Again, we replicated these analyses using a split-half approach, see Table S30. Although similar in effect size to the total sample (β’s are 0.013 and 0.009 for left amygdala functional connectivity vs. β = 0.010 in total sample) the indirect effect for left amygdala functional connectivity was significant in only 1 of the two subsamples.

The results of the sex-stratified analyses can be found in Table S23. For girls, a significant association was found for the indirect effects of left amygdala-cingulo-opercular network functional connectivity, β = 0.016, *p*_corr_ = 0.028. In boys, significant total and direct effects were found for left (β = 0.065, *p*_corr_ =0.005, β = 0.059, *p*_corr_ =0.012) and total effect for right amygdala functional connectivity (β = 0.048, *p*_corr_ =0.026).

### 2.3 Sensitivity analysis controlling for traumatic events

Results of the sensitivity analysis controlling for parent-reported traumatic events can be found in Table S31. Indirect effects for amygdala-cingulo-opercular network functional connectivity remained significant.

## 3 Discussion

Using data from the ABCD study, the present study aimed to replicate the findings on the mediating role of pubertal development in the relationship between a child’s family environment and structure and function of the amygdala-mPFC circuit as reported in Thijssen et al. (2020) in a large epidemiologically-informed independent sample, and to extend those findings using longitudinal data from the original sample. Results from the independent sample suggest that the direct effect of family environment and the mediating effect through pubertal stage could be replicated for amygdala-cingulo-opercular network intrinsic functional connectivity. The indirect effect for ACC cortical thickness remained significant but did not survive correction for multiple testing. Similar to Thijssen et al. (2020), analyses stratified for sex suggest that the indirect effects are significant for girls only. Similar to Thijssen et al. (2020), no associations between family environment or pubertal stage were reported with amygdala volume. However, while we do replicate direct associations between the family environment and ACC fractional anisotropy, the indirect effect of family environment on ACC fractional anisotropy via pubertal stage could not be replicated here. Finally, also the indirect quadratic mediation effect of pubertal stage on ACC cortical surface area could not be replicated. Longitudinal analyses using data from the originally-analyzed sample of more than 1800 youth confirm the resting-state fMRI direct and indirect effects, both when looking at functional connectivity at wave 2, as well as neurodevelopment from baseline to wave 2.

Although initial work on accelerated development of the amygdala-mPFC circuit focused on functional connectivity (Gee et al., 2013), Thijssen et al. (2020) hypothesized that development of structural modalities of the amygdala-mPFC circuit may also be accelerated. The results reported here indeed suggest that a more stressful family environment is associated with both structure and function of the amygdala-mPFC circuit in a manner consistent with accelerated development. The indirect effect via pubertal stage is most consistently found for the functional connectivity data, with less consistent evidence for ACC cortical thickness. We did not replicate indirect associations for ACC cortical surface area or fractional anisotropy. Gracia-Tabuenca et al. (2021) report that development of the functional connectome better fits pubertal development than chronological age. Although animal studies suggest effects of puberty on mPFC structure (Drzewiecki et al., 2016), two recent studies on puberty – cortical structure associations in human samples suggest that ACC structure is better predicted by age than pubertal stage (Ando et al., 2021, Vijayakumar et al., 2021a). This could explain why the mediating effect was found most consistently for the functional data, but not for ACC cortical surface area and inconsistently for ACC cortical thickness. Similarly, several prior studies report no or few associations between puberty and fractional anisotropy (Ando et al., 2021, Herting et al., 2017, Herting et al., 2012, Menzies et al., 2015).

Life history theories on accelerated pubertal development have focused mostly on girls (Ellis, 2004). As females are physically more restricted in the number of offspring they can conceive relative to males, it makes evolutionary sense that girls specifically mature sooner. Our results suggest that a stressful family environment accelerates pubertal development in both boys and girls, and is associated with neural structure and function in a manner consistent with accelerated development in both sexes. Nevertheless, sex stratified analyses point towards possible sex differences in the mechanism responsible for accelerated neural development. For girls, indirect effects via pubertal stage as well as direct effects of family environment on amygdala-mPFC structure and function were reported, suggesting that puberty as well as other mechanisms may be involved in accelerated neural development. Although analyses in the full sample point towards associations between pubertal stage and neural structure and function of the amygdala-mPFC circuit, when boys only were examined, pubertal stage was not associated with structure or function of the amygdala-mPFC circuit, and thus no evidence was found for accelerated puberty as a mechanism of accelerated neurodevelopment in boys alone. This is in line with studies suggesting that effects of pubertal hormones are smaller in boys than in girls (Ho et al., 2020, Juraska and Willing, 2017, Ladouceur et al., 2019, Vijayakumar et al., 2021b, Whittle et al., 2015). However, as pubertal development is slower in boys compared to girls, most boys in the current samples were still described as pre-pubertal at the study baseline (66 %/72 % in samples 1 and 2 vs 27 %/32 % of girls). Therefore, boys may have been too immature to capture associations between pubertal stage and neural development.

The longitudinal analyses suggest that, in boys, family environment, but not pubertal stage, is associated with function of the amygdala-mPFC circuit 2 years later, as well as with functional development from baseline to wave 2, in a manner consistent with accelerated neural development. For structure of the amygdala-mPFC circuit, only associations between family environment and wave 2 scores were found. For girls, pubertal stage, but not family environment at baseline was associated with function of the amygdala-mPFC circuit 2 years later as well as with change in functional data from baseline to wave 2. Whereas the results of the structural analyses provide evidence for a timing effect of family environment on neural development, for the functional data, both timing and tempo of neural development may be directly or indirectly affected by a more stressful family environment.

Interestingly, we did not find an association between family environment at baseline and pubertal tempo (pubertal change from baseline to wave 2, correcting for baseline pubertal stage). However, because of the age range of the participants, relatively few participants were in stage 4 or 5, even at wave 2. Therefore, this findings may be specific to early puberty and may not generalize to later pubertal development. Nevertheless, although the literature quite consistently reports associations between early environments and pubertal timing, fewer studies have examined associations with pubertal tempo. Relevant studies provide mixed results, with some suggesting that early stress is associated with increased pubertal tempo (Ellis et al., 2011, Negriff et al., 2015 (but only in boys)) or decreased pubertal tempo (Suglia et al., 2020), but most studies suggesting no association (Miller et al., 2020, Negriff et al., 2015, Saxbe et al., 2015).

The findings of both studies presented here as well as the study reported in Thijssen et al. (2020), were from large population-based samples, denoting reliability as well as generalizability. However, with larger sample size, smaller effects can become significant. While associations between family environment and pubertal stage are of moderate effect size, effect sizes of associations between pubertal stage and the neural outcomes are smaller. Therefore, any indirect effects reported here should be considered small. At the population level, however, even small effect sizes, such as those observed here, are potentially meaningful (Dick et al., 2021). Moreover, effect sizes of the associations between pubertal stage and amygdala-mPFC structure and function may increase with later releases, as more children reach pubertal stages 4 and 5.

Given that the current paper is a direct replication and extension of Thijssen et al. (2020), similar limitations apply here. These include the cross-sectional nature of study 1 and the relatively low levels of stress reported in this population-based sample. Of particular importance is a limitation concerning the family environment measure, which does not necessarily reflect early childhood family functioning as is commonly studied in other investigations of accelerated development (Gee et al., 2013; Thijssen et al., 2017). Because family functioning is reported to be stable (Loeber et al., 2000), and because our family environment variable includes other retrospective or stable demographic factors, we nevertheless believe that our measure of the child’s family environment constitutes an important predictor of pubertal and neural development. As discussed in Thijssen et al. (2020), ABCD did not release direct measures of amygdala-mPFC functional connectivity. Therefore, Thijssen et al. (2020) selected amygdala–cingulo-opercular functional connectivity as the best available proxy for amygdala–mPFC functional connectivity. However, the cingulo-opercular network only includes part of the mPFC as well as regions outside of the mPFC (i.e. insula).

In conclusion, the current paper replicates the findings of a mediating role for pubertal stage in the association between a child’s family environment and amygdala-mPFC function, but not structure. As both direct and indirect effects of the family environment on the amygdala-mPFC circuit were found, pubertal development may be one of the mechanisms driving accelerated neurodevelopment in response to normative variation in familial stress. This mechanism may be especially relevant for functional connectivity (and possibly cortical thickness development) for girls, whereas for other modalities and for boys, other mechanisms may be at play. Future studies using longitudinal data, preferably starting at an even younger age, should shed further light on the role of pubertal development in accelerated neural development and should examine the effects of pubertal hormones.

## Declaration of Competing Interest

The authors declare that they have no known competing financial interests or personal relationships that could have appeared to influence the work reported in this paper.
