# Supplementary material for: Does pubertal stage mediate the association between family environment and structure and function of the amygdala-mPFC circuit? A replication study of the longitudinal ABCD cohort
Source: Dev Cogn Neurosci. 2022 Jun 10;56:101120. doi: 10.1016/j.dcn.2022.101120 (PMC9213703; doi:10.1016/j.dcn.2022.101120)
Supplement: Supplementary file 1 — Supplementary material. [file mmc1.docx]

**Does pubertal stage mediate the association between family environment and structure and function of the amygdala-mPFC circuit? A replication study of the longitudinal ABCD cohort**

Sandra Thijssen^1,2,3^, Paul F. Collins^3^, Monica Luciana^3^

**Supplemental material.**

**Contents**

[**Figure S1**. Flowchart study 1 5](#_Toc94188200)

[**Figure S2**. Flowchart study 2 6](#_Toc94188201)

[**Text S1. Family Environment Variable** 7](#_Toc94188202)

[**Figure S3a**.Factor loadings structural equation model Study 1. 9](#_Toc94188203)

[**Figure S3b**.Factor loadings structural equation model Study 2. 10](#_Toc94188204)

[Table S1. Partial correlations between LVs 11](#_Toc94188205)

[**Distribution of pubertal stage** 12](#_Toc94188206)

[Table S2a. *Distribution of pubertal stage: total sample* 12](#_Toc94188207)

[Table S2b. *Distribution of pubertal stage: MRI T1 sample* 12](#_Toc94188208)

[Table S2c *Distribution of pubertal stage: DTI sample* 13](#_Toc94188209)

[Table S2d *Distribution of pubertal stage: rs-fMRI sample* 13](#_Toc94188210)

[**Supplemental Text 2. MRI acquisition and preprocessing** 14](#_Toc94188211)

[**Preprocessing and MRI data analysis** 15](#_Toc94188212)

[**Brain structure by pubertal stage** 20](#_Toc94188213)

[Figure S4. Brain structure by pubertal stage Study 1 20](#_Toc94188214)

[. Figure S5. Brain structure by pubertal stage Study 2 21](#_Toc94188215)

[**Supplemental Text 3. Association between family environment and menarche** 22](#_Toc94188216)

[**Results Study 1** 23](#_Toc94188217)

[Table S3a. Sample characteristics; continuous variables 23](#_Toc94188218)

[Table S3b. Sample characteristics; categorical variables 24](#_Toc94188219)

[Table S4*. Sex-corrected correlations between outcome-variables Study 1* 25](#_Toc94188220)

[Table S5. *Total, direct, and indirect family environment on amygdala-mPFC structure and function of stratified analyses in Study 1* 26](#_Toc94188221)

[Table S6. *Mediation model parameters – anterior cingulate cortical thickness and area, and amygdala volume Study 1* 28](#_Toc94188222)

[Table S7. *Mediation model parameters – anterior cingulate cortical thickness and surface area and amygdala volume Study 1 in girls* 29](#_Toc94188223)

[Table S8. *Mediation model parameters – anterior cingulate cortical thickness and surface area and amygdala volume Study 1 in boys* 30](#_Toc94188224)

[Table S9. *Mediation model parameters – cinculo-opercular network-amygdala connectivity* 31](#_Toc94188225)

[Table S10*. Mediation model parameters – cinculo-opercular network-amygdala connectivity in girls* 32](#_Toc94188226)

[Table S11. *Mediation model parameters – cinculo-opercular network-amygdala connectivity in boys* 33](#_Toc94188227)

[Table S12. *Mediation model parameters – anterior cingulate white matter fractional anisotropy* 34](#_Toc94188228)

[Table S13. *Mediation model parameters – anterior cingulate white matter fractional anisotropy in girls* 35](#_Toc94188229)

[Table S14. *Mediation model parameters – anterior cingulate white matter fractional anisotropy in boys* 35](#_Toc94188230)

[**Results Study 2. Wave 2 analyses** 36](#_Toc94188231)

[Table S15. *Sex-corrected correlations between outcome-variables wave 2* 36](#_Toc94188232)

[Table S16. *Total, direct, and indirect family environment on amygdala-mPFC structure and function of stratified analyses in wave 2* 37](#_Toc94188233)

[Table S17. *Mediation model parameters – anterior cingulate cortical thickness and area, and amygdala volume wave 2* 38](#_Toc94188234)

[Table S18. *Mediation model parameters – anterior cingulate cortical thickness and surface area and amygdala volume in girls* 39](#_Toc94188235)

[Table S19. *Mediation model parameters – anterior cingulate cortical thickness and surface area and amygdala volume in boys* 40](#_Toc94188236)

[Table S20. *Mediation model parameters – cinculo-opercular network-amygdala connectivity study 2* 41](#_Toc94188237)

[Table S21. *Mediation model parameters – cinculo-opercular network-amygdala connectivity in girls* 42](#_Toc94188238)

[Table S22. *Mediation model parameters – cinculo-opercular network-amygdala connectivity in boys* 43](#_Toc94188239)

[**Study 2. Neural change analyses** 44](#_Toc94188240)

[**Supplemental Text 4. Wave 2 analyses** 44](#_Toc94188241)

[*Structural MRI* 44](#_Toc94188242)

[Table S23. *Total, direct, and indirect family environment on amygdala-mPFC structure and function of stratified wave 2-wave 1 difference scores* 45](#_Toc94188243)

[Table S24. *Mediation model parameters – anterior cingulate cortical thickness and surface area and amygdala volume wave 2-wave 1 change in total sample* 46](#_Toc94188244)

[Table S25. *Mediation model parameters – anterior cingulate cortical thickness and surface area and amygdala volume wave 2-wave 1 change in girls* 47](#_Toc94188245)

[Table S26. *Mediation model parameters – anterior cingulate cortical thickness and surface area and amygdala volume wave 2-wave 1 change in boys* 48](#_Toc94188246)

[Table S27. *Mediation model parameters – cinculo-opercular network-amygdala connectivity wave 2-wave 1 change in total sample* 49](#_Toc94188247)

[Table S28. *Mediation model parameters – cinculo-opercular network-amygdala connectivity wave 2-wave 1 change in girls* 50](#_Toc94188248)

[Table S29. *Mediation model parameters – cinculo-opercular network-amygdala connectivity wave 2-wave 1 change in boys* 51](#_Toc94188249)

[Table S30. *Split half-replication of rs-fMRI analyses in Study 2: total, direct, and indirect family environment on amygdala-mPFC functional connectivity* 52](#_Toc94188250)

[References 53](#_Toc94188251)

# **Figure S1**. Flowchart study 1

Total sample

N = 11.875

Total sample

N = 11.314

Missing biological parent data

N = 561

MRI incidental findings

N = 335

Twins and siblings

N = 2779

Participants of original study

N = 3001

Total sample

N = 10.979

Total sample

N = 8200

Family environment sample

N = 5199

Available T1w data (release 3.0)

N = 5023

Available DTI data (release 2.0.1)

N = 4472

Available rs-fMRI

(release 3.0) data

N = 4796

Poor or moderate quality T1w data

N = 707

FD > 0.55 mm

N =628

< 4 min data with FD < 0.20 mm

N = 276

Final T1w sample

N = 4316

Final DTI sample

N = 3359

Final rs-fMRI sample

N = 3892

FD > 1.50 mm

N = 1113

# **Figure S2**. Flowchart study 2

Total sample release 1

N = 4524

N = 3553

Twins and siblings

N = 971

Missing biological parent data

N = 194

MRI incidental findings

N = 176

N = 3359

Family environment sample

N = 3183

Available wave 1 T1w data

N = 3048

Available wave 1 rs-fMRI data

N = 2727

Poor or moderate quality T1w data

N = 553

FD > 0.55 mm
N =271
< 4 min data with FD < 0.20 mm
N = 50

Final T1w sample Thijssen et al. 2020

N = 2495

Final rs-fMRI sample Thijssen et al. 2020

N = 2461

Available wave 2 T1w data (release 3.0)

N = 2016

Poor or moderate quality T1w data

N = 138

Final wave 2 T1w data

N = 1878

Available wave 2 rs-fMRI data (release 3.0)

N = 1977

FD > 0.55 mm
N =47
< 4 min data with FD < 0.20 mm
N = 5

Final wave 2 rs-fMRI data

N = 1925

# **Text S1. Family Environment Variable**

For a detailed description and rationale for the measures collected in the ABCD study that may be relevant for the family environment construct, please see Barch et al. (2018) for demographic, physical and mental health assessments, and Zucker et al. (2018) for the assessment of culture and environment.

In order to create a latent measure reflecting the quality of family environment, three types of information were used: child reported information about family dynamics and relationships, parent reported information about family dynamics and relationships, and demographic and parent information. Child reported questionnaires included an abbreviated version of the Maternal Acceptance scale of the Child Report of Parent Behavior Inventory (CRPBI, Schaefer, 1965), the Conflict scale from the Family Environment Scale (Moos & Moos, 1976), and the Parental Monitoring Survey (Chilcoat & Anthony, 1996). The abbreviated version of the Maternal Acceptance scale of the CRPBI assesses maternal acceptance versus rejection and consists of five items (e.g. ‘smiles at me very often’) measured on a 3-point Likert scale ranging from ‘not like her’ to ‘a lot like her’. The Parental Monitoring Survey consists of 5 items answered on a 5-point Likert scale ranging from ‘never’ to ‘always or almost always’ (e.g. ‘how often do your parents know where you are?’), and assesses whether the child believes that his/her parent knows of their whereabouts and activities. The Conflict scale of the FES consists of 9 true or false item that aim to measure conflict within the family (e.g. ‘we fight a lot in our family’). The parent reported information also included the Conflict scale from the Family Environment Scale, and was complimented by one background item from the Kiddie Schedule for Affective Disorders and Schizophrenia (KSADS, Kaufman et al., 1997) measuring the relationship between the parent and the child (‘in general, how do you and your child get along?’, using a 3 point Likert scale ranging from ‘very well’ to ‘a lot of conflict’). Contrary to the Thijssen et al. (2020) study, where we added parental psychopathology to the demographics variable, parental psychopathology (total problem score of the ASEBA Adult Self Report, Achenbach & Rescorla, 2003)was added to the parent latent variable. Demographic and parent information included family yearly income (rated on a 10-point scale ranging from ‘less than 5000’ to ‘200.000 or more’), parental education (6-point scale ranging from ‘finished high-school or less’ to ‘professional school or doctoral degree’, PhenX, Stover et al., 2010), , parental relationship status (i.e. are biological parents still together?), and the planned nature of the pregnancy (i.e. did parents plan this pregnancy,Kessler et al., 2009).

The child-reported (STD standardized loading = 0.553, and 0.578, for Study 1 and 2, respectively), parent-reported (STD standardized loading = 0.531, and 0.507, for Study 1 and 2, respectively), and demographical latent variables (STD standardized loading = 0.451, and 0.359, for Study 1 and 2, respectively) were combined to yield an overall family environment latent variable (see Supplemental Figure S3a and b). The model had acceptable fit, root mean square error of approximation = .036, and .033 , Study 1 and 2, respectively, 95% confidence interval (CI) [.035, .037] and [.031, .033], Study 1 and 2, respectively, comparative fit index = .90, and .91, Study 1 and 2, respectively, and Tucker–Lewis index = .89, and .90, for Study 1 and 2, respectively.

#


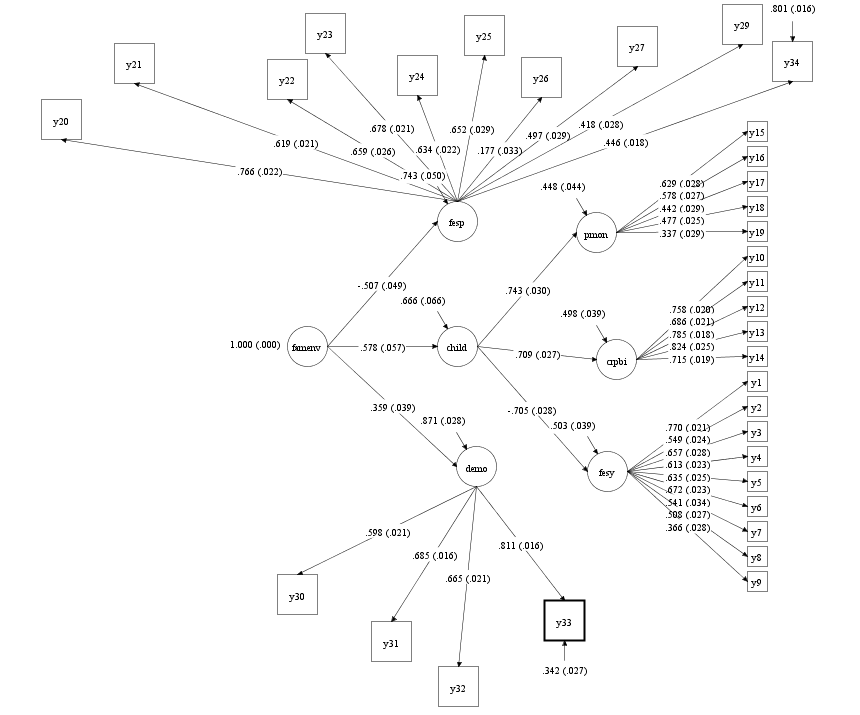


P

D

C

FE

# **Figure S3a**.Factor loadings structural equation model Study 1.

FE = Family Environment; P = Parent-reported latent variable; C = Child-report latent variable; D = Demographic and parental latent variable; pmon = Parental monitoring latent variable; crpbi = Child report of parent behavior inventory latent variable; fesy = Family Environment Scale youth report latent variable; y1-y9 = items of Conflict Scale of FESY; y10-y14 = Items of Parental acceptance scale of CRPBI; y15-y19 = items of Parental Monitory Survey; y20-y25, y27 = Items of Conflict scale of FES parent report; y29 = KSADS item on parent-child conflict; y30 = planned pregnancy; y31 = parental education; y32 = parental separation; y33 = family income; y34 = parental psychopathology


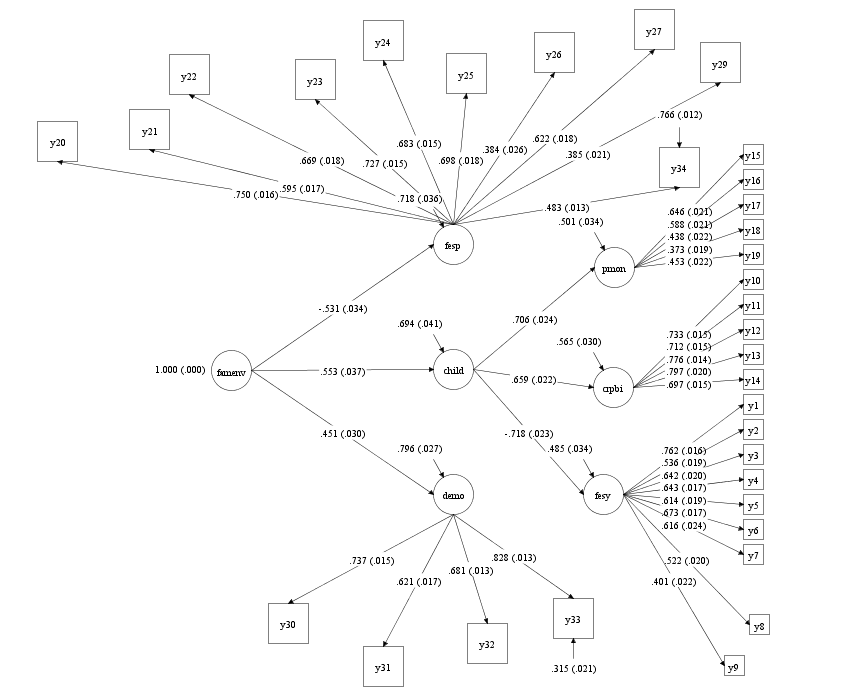
.

C

P

FE

D

# **Figure S3b**.Factor loadings structural equation model Study 2.

FE = Family Environment; P = Parent-reported latent variable; C = Child-report latent variable; D = Demographic and parental latent variable; pmon = Parental monitoring latent variable; crpbi = Child report of parent behavior inventory latent variable; fesy = Family Environment Scale youth report latent variable; y1-y9 = items of Conflict Scale of FESY; y10-y14 = Items of Parental acceptance scale of CRPBI; y15-y19 = items of Parental Monitory Survey; y20-y25, y27 = Items of Conflict scale of FES parent report; y29 = KSADS item on parent-child conflict; y30 = planned pregnancy; y31 = parental education; y32 = parental separation; y33 = family income; y34 = parental psychopathology

# Table S1. Partial correlations between LVs

|  | Study 1 | | | Study 2 | | |
| --- | --- | --- | --- | --- | --- | --- |
|  | Child LV | Parent LV | Demo-  graphics | Child LV | Parent LV | Demo-graphics |
| FE LV | .780 | .774 | .693 | .820 | .672 | .578 |
| Child LV |  | .396 | .343 |  | .383 | .278 |
| Parent LV |  |  | .293 |  |  | .223 |

Note. Correlations were corrected for child age and sex. FE = Family Environment latent variable; Child LV = latent variable representing child-reported data on parenting and family relationships; Parent LV = latent variable representing parent-reported data on family relationships; Demographics = latent variable representing family demographical information

# **Distribution of pubertal stage**

## Table S2a. *Distribution of pubertal stage: total sample*

|  | Study 1 | | | Study 2 | | |
| --- | --- | --- | --- | --- | --- | --- |
| Pubertal stage | Total sample (n=5199) | Boys (n=2712) | Girls (n=2485) | Total sample (n=3183) | Boys (n=1725) | Girls (n=1458) |
| 1 | 2441 (47.0) | 1773 (65.4) | 667 (26.8) | 1697 (53.3) | 1234 (71.5) | 463 (31.8) |
| 2 | 1283 (24.7) | 687 (25.3) | 596 (24.0) | 765 (24.0) | 400 (23.2) | 365 (25.0) |
| 3 | 1294 (24.9) | 194 (7.2) | 1099 (44.2) | 664 (20.9) | 83 (4.8) | 581 (39.8) |
| 4 | 89 (1.7) | 19 (0.7) | 70 (2.8) | 42 (1.3) | 8 (0.5) | 34 (2.3) |
| 5 | 8 (0.2) | 4 (0.1) | 4 (0.2) | 0 (0.0) | 0 (0.0) | 0 (0.0) |

## Table S2b. *Distribution of pubertal stage: MRI T1 sample*

|  | Study 1 | | | Study 2 | | |
| --- | --- | --- | --- | --- | --- | --- |
| Pubertal stage  wave 1 | Total sample (n=4316) | Boys (n=2218) | Girls (n=2096) | Total sample (n=1878) | Boys (n=1003) | Girls (n=875) |
| 1 | 2027 (47.0) | 1464 (66.0) | 562 (26.8) | 1023 (54.5) | 712 (71.0) | 311 (35.5) |
| 2 | 1052 (24.4) | 544 (24.5) | 508 (24.2) | 447 (23.8) | 244 (24.3) | 203 (23.2) |
| 3 | 1089 (25.2) | 160 (7.2) | 928 (44.3) | 382 (20.3) | 44 (4.4) | 338 (38.6) |
| 4 | 75 (1.7) | 18 (0.8) | 57 (2.7) | 19 (1.0) | 3 (0.3) | 16 (1.8) |
| 5 | 6 (0.1) | 3 (0.1) | 3 (0.1) | 0 (0.0) | 0 (0.0) | 0 (0.0) |

## Table S2c *Distribution of pubertal stage: DTI sample*

| Pubertal stage | Total sample (n= 3360) | Boys (n=1709) | Girls (n=1650) |
| --- | --- | --- | --- |
| 1 | 1600 (47.6) | 1148 (67.2) | 451 (27.3) |
| 2 | 829 (24.7) | 419 (24.5) | 410 (24.8) |
| 3 | 822 (24.5) | 107 (6.3) | 715 (43.3) |
| 4 | 53 (1.6) | 10 (0.6) | 43 (2.6) |
| 5 | 3 (0.1) | 1 (0.1) | 2 (0.1) |

## Table S2d *Distribution of pubertal stage: rs-fMRI sample*

|  | Study 1 | | | Study 2 | | |
| --- | --- | --- | --- | --- | --- | --- |
| Pubertal stage | Total sample (n=3476) | Boys (n=1752) | Girls (n=1722) | Total sample (n=1925) | Boys (n=1036) | Girls (n=889) |
| 1 | 1634 (47.0) | 1174 (67.0) | 459 (27.6) | 1068 (55.5) | 762 (73.6) | 306 (34.4) |
| 2 | 858 (24.6) | 432 (24.7) | 422 (24.5) | 452 (23.5) | 227 (21.9) | 225 (25.3) |
| 3 | 878 (25.3) | 116 (6.6) | 761 (44.2) | 377 (19.6) | 42 (4.1) | 335 (37.7) |
| 4 | 60 (1.7) | 12 (0.7) | 48 (2.8) | 19 (1.0) | 5 (0.5) | 14 (1.6) |
| 5 | 3 (0.1) | 1 (0.1) | 2 (0.1) | 0 (0.0) | 0 (0.0) | 0 (0.0) |

# **Supplemental Text 2. MRI acquisition and preprocessing**

For details on MRI acquisition in the ABCD study, please see Casey et al. (2018). Across all sites, participants were familiarized to the MRI environment using a mock scanner. A two-hour MRI scanning session was performed on different 3T scanners from Siemens (Siemens Medical Systems, Erlangen, Germany), Philips (Philips Medical Systems, Best, the Netherlands), as well as GE (General Electric, Milwaukee, MI, USA). A T1-weighted anatomical scan was acquired as follows: Siemens scanners: TR = 2500 ms, TE = 2.88 ms, flip angle = 8°, 176 transverse slices, voxel size 1 × 1 × 1 mm; Philips scanners: TR = 6.31 ms, TE = 2.9 ms, flip angle = 8°, 255 transverse slices, voxel size 1 × 1 × 1 mm; GE scanners: TR = 2500 ms, TE = 2.0 ms, flip angle = 8°, 208 transverse slices, voxel size 1 × 1 × 1 mm).

Diffusion tensor imaging data were acquired using a multiband, echo-planar imaging sequence with the following parameters: TR = 4100 ms Siemens and GE/5300 ms Philips, TE = 88 ms Siemens/81 ms Philips/89 ms GE, flip angle = 90 Siemens/78 Philips/ 77 GE, matrix = 140 × 140, FOV = 240 mm × 240 mm, slice thickness = 1.7 mm, number of slices = 81, 96 diffusion directions, 7 b=0 frames, 4 b-values (6 directions with b=500s/mm^2^, 15 directions with b=1000s/mm^2^, 15 directions with b=2000s/mm^2^, 60 directions with b=3000s/mm^2^), acquisition time = 7 min 31 s Siemens/9 min 14 s Philips/7 min 30 s GE.

The resting-state fMRI-sequence utilized the same gradient-echo blood oxygen level dependent (BOLD) EPI sequence for each scanner with: TR = 800 ms, TE = 30 ms, flip angle = 52°, 60 transverse slices, multiband acceleration = 6, and voxel resolution of 2.4 × 2.4 × 2.4 mm. Three to four runs of five minutes were acquired. The first two runs as well as run three and four were separated by a 10s film clip. The second and the third run were separated by a diffusion scan.

## **Preprocessing and MRI data analysis**

MRI preprocessing and analyses were performed by the ABCD consortium’s data analytic core. Please see (Hagler et al., 2019) for detailed information.

T1-weighted images were corrected for gradient nonlinearity distortions (Jovicich et al., 2006). T2-weighted images were registered to T1-weighted images using mutual information (Wells et al., 1996). Intensity non-uniformity correction was performed based on tissue segmentation and sparse spatial smoothing. Data were resampled with 1 mm isotropic voxels into rigid alignment with an atlas brain. Cortical surface reconstruction was performed using FreeSurfer v5.3.0 (<https://surfer.nmr.mgh.harvard.edu>). Briefly, FreeSurfer was used for skull-stripping (Segonne et al., 2004), white matter segmentation, initial mesh creation (Dale et al., 1999), correction of topological defects (Fischl et al., 2001; Segonne et al., 2007), surface optimization (Dale et al., 1999; Dale & Sereno, 1993; Fischl & Dale, 2000), and nonlinear registration to a spherical surface-based atlas (Fischl et al., 1999). Subcortical structures were labeled with atlas-based segmentation (Fischl et al., 2002).

For the diffusion MRI images, eddy current correction uses a model-based approach, predicting the pattern of distortions across the entire set of diffusion weighted volumes, based on diffusion gradient orientations and amplitudes, with corrections limited to displacement along the phase-encode direction (Zhuang et al., 2006). During each iteration, a robust tensor fit was calculated in which data frames with high residual error were excluded from the linear estimation of tensor model parameters. Outlier data frames, e.g., slices showing signal dropout due to sudden head movements, were replaced with values estimated from the tensor fit based on the censored data. More subtle forms of head motion were corrected using rigid body registration of each data frame with the corresponding eddy current corrected volume (Hagler et al., 2009). The diffusion gradient matrix was adjusted for head rotation (Hagler et al., 2019; Leemans & Jones, 2009) and mean head motion values were calculated to correct for residual motion effects in group statistical analyses.

Spatial and intensity distortions caused by B_0_ field inhomogeneity were reduced using the reversing gradient method (Holland et al., 2010). Pairs of b=0 (non-diffusion weighted) images with opposite phase encoding polarities were aligned using a nonlinear registration procedure, and the estimated displacement field volume was used to correct distortions in each successive diffusion-weighted volume. To use anatomical ROIs from FreeSurfer’s automated subcortical segmentation and cortical parcellation, b=0 images were registered to T_1_ weighted structural images using mutual information (Wells et al., 1996) after coarse pre-alignment via within-modality registration to atlas brains. Diffusion images were resampled into a standard orientation with 1.7 mm isotropic voxel resolution. Standard-space registration was combined with the motion-correction registration, so that a single resampling step could be performed using cubic interpolation.

Several standard measures related to microstructural tissue properties were calculated after fitting the diffusion tensor, including fractional anisotropy, and mean, longitudinal (or axial), and transverse diffusivity (MD, LD, and TD). B values greater than 1000 were excluded from tensor fitting to avoid need for nonlinear estimation, and diffusion tensor parameters were calculated using a linear estimation approach with log-transformed diffusion-weighted (DW) signals (Basser et al., 1994).

Mean DTI measures were calculated for ROIs derived from FreeSurfer’s automated segmentation and parcellation. To minimize partial volume effects, mean DTI measures for cortical ROIs were weighted based on the proportion of white versus gray matter within each voxel in an ROI, using information from the cortical surfaces generated by FreeSurfer during processing of T_1_ weighted images. A similar method was used to calculate weighted mean mean diffusivity (MD) values for subcortical ROIs, to minimize signal contamination due to CSF partial voluming within voxels (Elman et al., 2017).

Resting-state fMRI data were head motion corrected by registering each frame to the first using AFNI’s 3dvolreg (Cox, 1996). B0 distortions were corrected using the reversing gradient method (Holland et al., 2010). Displacement field was estimated from spin-echo field map scans, then adjusted for estimated between-scan head motion, and applied to gradient-echo images. Data were corrected for gradient nonlinearity distortions (Jovicich et al., 2006). Finally, between scan motion correction was performed across all fMRI scans in imaging session Registration was performed between T2-weighted, spin-echo B0 calibration scans and T1-weighted structural images using mutual information(Wells et al., 1996)**.**

Following initial preprocessing, initial volumes were removed from the scan (Siemens/Philips: 8 TRs, GE DV25: 5 TRs, GE DV26: 16 TRs). Data were normalized and demeaned. Then, linear regression was performed to remove quadratic trends and signals correlated with motion and mean time courses of cerebral white matter, ventricles, and whole brain, plus first derivatives (Power et al., 2014; Satterthwaite et al., 2012) . Motion regression included six parameters plus their derivatives and squares. Frames with displacement > 0.3 mm were excluded from the regression (Power et al., 2014)**.** Motion estimates were filtered to attenuate signals (0.31 - 0.43 Hz) associated with respiration (18.6 - 25.7 respirations / minute). After regression, data were band-pass filtered between 0.009 and 0.08 Hz (Hallquist et al., 2013).

Preprocessed time courses were sampled onto the cortical surface projecting 1mm into cortical gray matter along surface normal vector. Motion censoring was performed to reduce residual effects of head motion (Power et al., 2012; Power et al., 2014). ime points with FD > 0.2 mm were excluded from variance and correlation calculations as well as time periods with < 5 contiguous, sub-threshold time points and time points that were outliers in standard deviation across ROIs. Subcortical structures were labeled with atlas-based FreeSurfer segmentation (Fischl et al., 2002). Networks were defined as predefined groups of parcels stemming from functionally-defined parcellation based on resting-state functional connectivity patterns (Gordon et al., 2016). A seed-based, correlational approach (Van Dijk et al., 2010) adapted for cortical surface based analysis (Seibert & Brewer, 2011) was performed. The average correlation between networks and ROIs is calculated as the average of the Fisher-transformed correlations.

The present study made use of tabulated datasheets resulting from these analyses. Because we had no hypotheses related to laterality effects, for all ACC modalities, left and right, rostral and caudal ACC measures were averaged to create a summary ACC score. To create an average amygdala volume score, right and left amygdala volumes were averaged.

Quality assurance

Study 1: Cross-sectional replication

FreeSurfer-processed structural data were available on 5023 participants. We excluded 707 participants whose data sets were rated by the ABCD consortium as moderately or severely impacted by motion, intensity inhomogeneity, white matter underestimation, pial overestimation, or magnetic susceptibility artifact, resulting in 4316 participants.

Processed DTI data were available on 4472 participants. We excluded 1113 participants with more than 1.50 mm average framewise displacement, resulting in a total sample of 3359 participants.

For the resting-state analyses, fully processed data were available on 4796 participants. We further excluded 628 participants with a mean FD of >0.55 mm as well as 276 participants who had less than 4 min of resting-state data with FD < 0.20 mm (Parkes et al., 2018).

Study 2: Longitudinal Analysis

FreeSurfer-processed structural data were available on 2016 participants of the original 2495 participants. We excluded 138 participants whose data sets were rated by the ABCD consortium as moderately or severely impacted by motion, intensity inhomogeneity, white matter underestimation, pial overestimation, or magnetic susceptibility artifact, resulting in 1878 participants.

For the resting-state analyses, fully processed data were available on 1977 of the original 2461 participants. We excluded 47 participants with a mean FD of >0.55 mm as well as 5 participants who had less than 4 min of resting-state data with FD < 0.20 mm (Parkes et al., 2018).

# **Brain structure by pubertal stage**


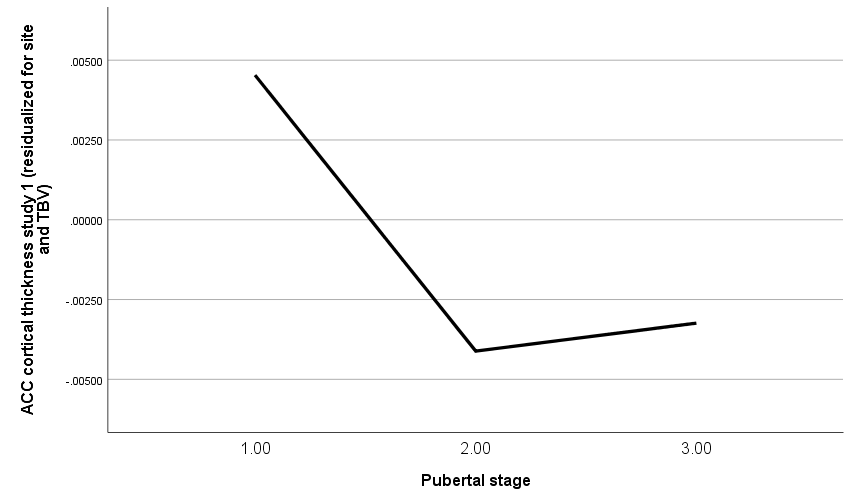

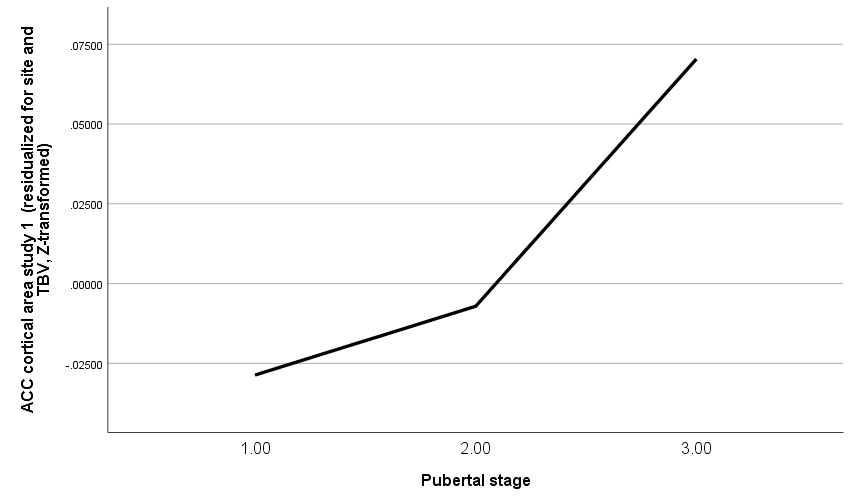

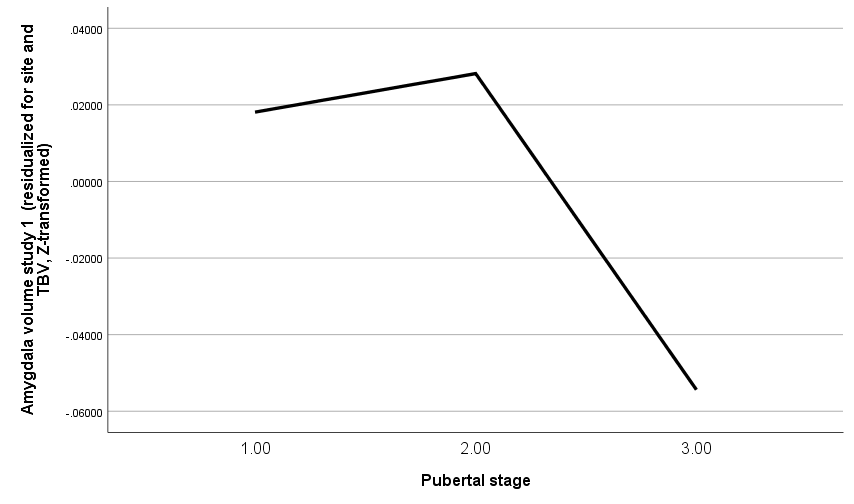


## Figure S4. Brain structure by pubertal stage Study 1


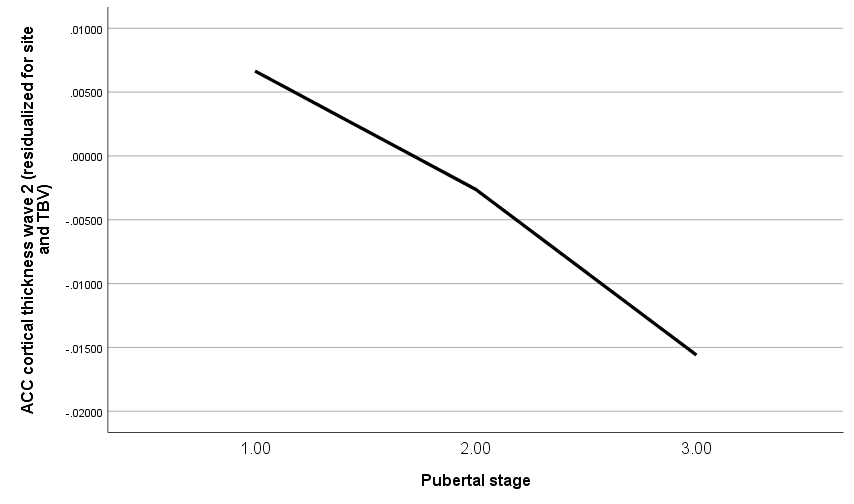

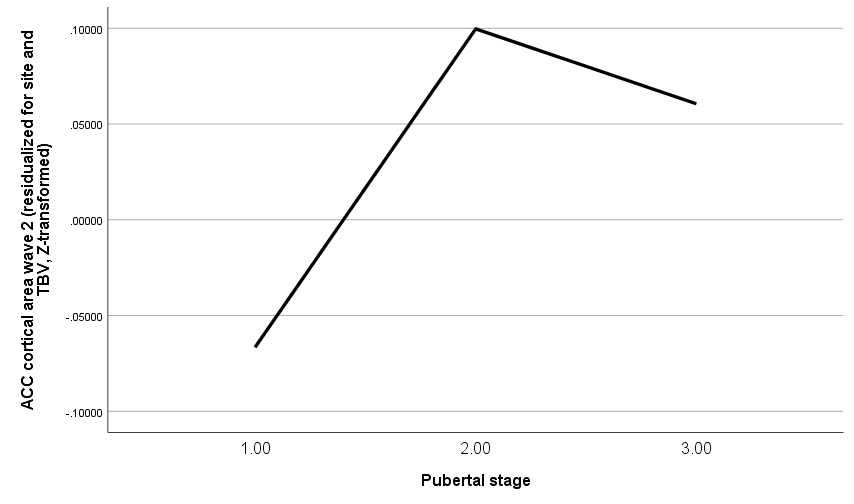

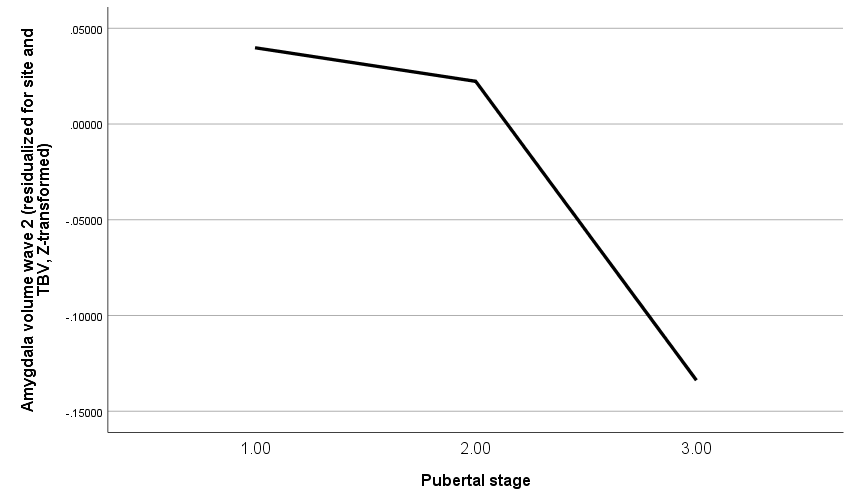

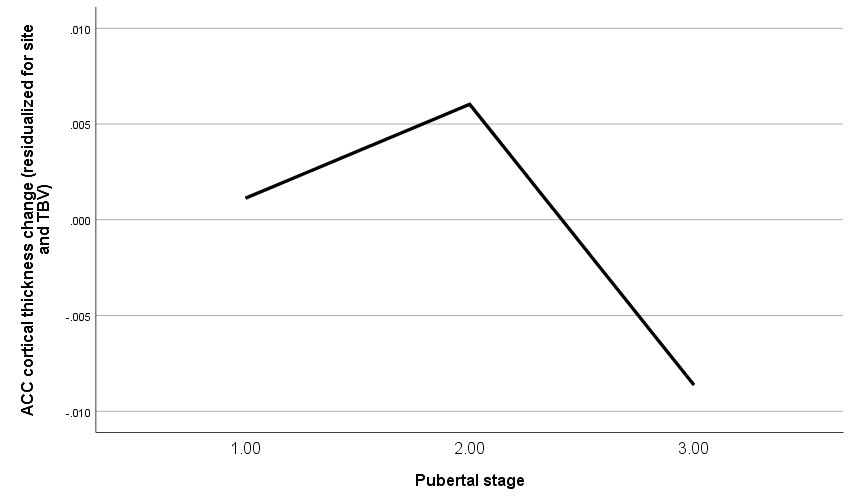

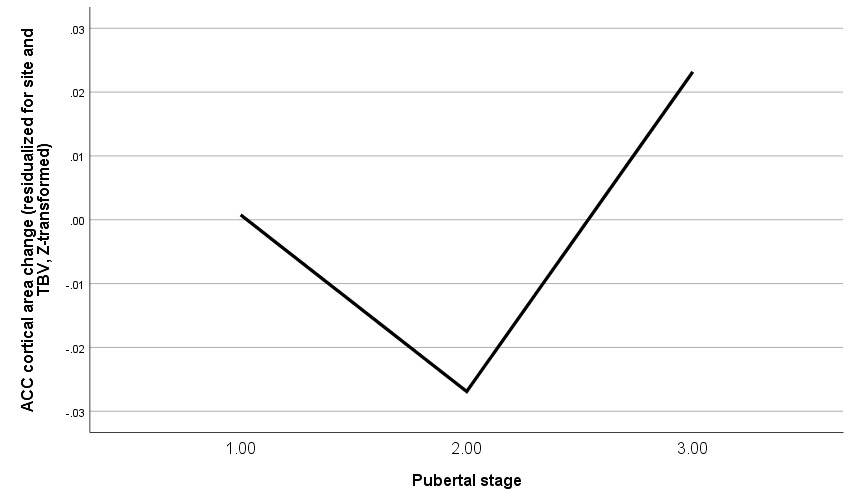

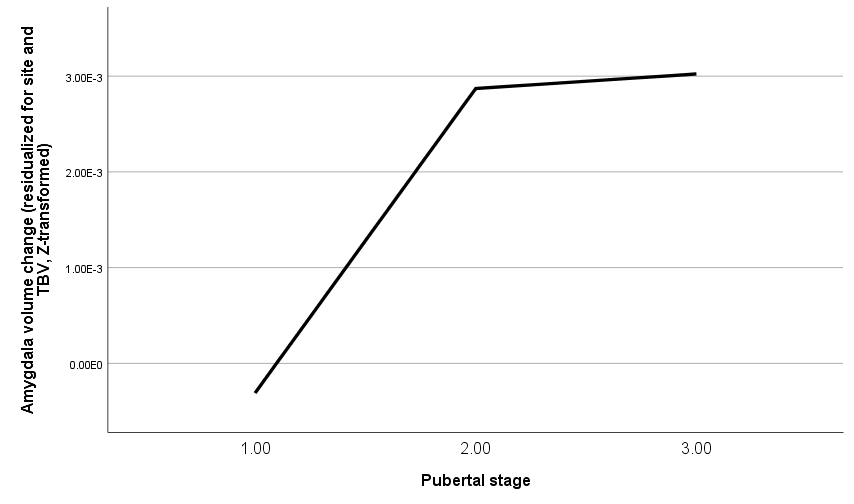


A

B

## . Figure S5. Brain structure by pubertal stage Study 2

A. Wave 2 B. Wave 2 – Wave 1 difference scores

# **Supplemental Text 3. Association between family environment and menarche**

In order to examine whether family environment was associated with the experience of menarche in girls, binary logistic regression analyses were performed, correcting for age and ethnicity. In the sample of study 1, 57 of 1926 girls had experienced menarche at wave 1. Family environment was a significant predictor of menarche, OR = 0.443, *p* < .001. In the study 2 sample, 26 of 1146 girls experienced menarche at wave 1. Family environment was a significant predictor of menarche, OR = 0.380, *p* = .007.

We further performed linear regression analyses to examine the association between family environment and age at menarche at wave 2 (n = 221 for study 1, n = 370 for study 2). After correction for age and ethnicity, family environment was significantly associated with age at menarche, β = .134, p = .034 and β = .092, p = .062, for study 1 and 2, respectively.

# **Results Study 1**

|  | Study 1 | | | | Study 2 | | | |
| --- | --- | --- | --- | --- | --- | --- | --- | --- |
|  | N | *M* | *S.D.* | Range | N | *M* | *S.D.* | Range |
| Age (months) | 5199 | 118.18 | 7.52 | 108-131 | 3183 | 119.42 | 7.25 | 107 – 132 |
| Parental Acceptance | 5176 | 2.78 | 0.31 | 1.00-3.00 | 3178 | 2.79 | 0.29 | 1.00 – 3.00 |
| Parental Monitoring | 5181 | 4.37 | 0.52 | 1.60-5.00 | 3182 | 4.41 | 0.50 | 2.00 – 5.00 |
| Family Conflict child | 5181 | 2.03 | 1.95 | 0.00-9.00 | 3181 | 1.94 | 1.89 | 0.00 – 9.00 |
| Family Conflict parent | 5180 | 2.57 | 2.01 | 0.00-9.00 | 3171 | 2.46 | 1.84 | 0.00 – 9.00 |
| Parental psychopathology | 5198 | 21.88 | 18.85 | 0.00-154.00 | 3114 | 37.58 | 17.19 | 0.00 – 142.00 |
| Income | 4728 | 6.97 | 2.57 | 1.00-10.00 | 2907 | 7.37 | 2.28 | 1.00 – 10.00 |
| Family Environment LV | 5199 | -0.05 | 0.62 | -2.29 – 1.53 | 3183 | -0.06 | 0.56 | -2.23 – 1.41 |
| Child reported LV | 5199 | -0.08 | 0.81 | -3.23 – 1.66 | 3183 | -0.10 | 0.89 | -3.35 – 1.75 |
| Parent reported LV | 5199 | 0.06 | 0.96 | -1.97 – 3.17 | 3183 | 0.05 | 0.91 | -1.77 –3.78 |
| Demographics LV | 5199 | -0.03 | 1.03 | -4.06 – 2.09 | 3183 | -0.03 | 0.92 | -2.79 – 1.62 |

## Table S3a. Sample characteristics; continuous variables

Note. LV = latent variable

## Table S3b. Sample characteristics; categorical variables

|  | | Study 1 | | Study 2 | |
| --- | --- | --- | --- | --- | --- |
| Categorical variable | | N | *N(%)* | N | *N(%)* |
| Boys |  | 5199 | 2712 (52.2) | 3183 | 1725 (54.19) |
| Race | Caucasian | 5189 | 2417 (46.5) | 1797 | 1797 (56.46) |
|  | Black |  | 910 (17.5) |  | 285 (8.95) |
|  | Hispanic |  | 1180 (22.7) |  | 724 (22.74) |
|  | Other |  | 682 (13.1) |  | 377 (11.85) |
| Pubertal stage | 1 | 5115 | 2441 (47.0) | 3168 | 1697 (53.31) |
|  | 2 |  | 1283 (24.7) |  | 765 (24.03) |
|  | 3+ |  | 1391 (26.8) |  | 706 (22.18) |
| Parental education | High school or less | 5199 | 101 (20.0) | 3138 | 495 (15.55) |
|  | Some college |  | 875 (16.8) |  | 515 (16.18) |
|  | Associate degree |  | 688 (13.2) |  | 393 (12.35) |
|  | Bachelor’s degree |  | 1344 (25.9) |  | 945 (29.69) |
|  | Master’s degree |  | 940 (18.1) |  | 625 (19.64) |
|  | Professional School/Doctorate degree | | 311 (6.0) |  | 210 (6.60) |
| Parental divorce/separation | | 5124 | 1801 (34.7) | 3151 | 981 (30.82) |
| Planned pregnancy | | 5163 | 3081 (59.3) | 3158 | 2010 (63.15) |

## Table S4*. Sex-corrected correlations between outcome-variables Study 1*

|  | ACC CA | Amygdala SV | ACC FA | CON-l amygdala FC | CON-r amygdala FC | Age^$^ |
| --- | --- | --- | --- | --- | --- | --- |
| ACC CT | -.08 | .06 | -.08 | .01 | .00 | -.12 |
| ACC CA |  | -.03 | -.34 | -.01 | -.00 | .02 |
| Amygdala SV |  |  | -.00 | .02 | -.01 | .03 |
| ACC FA |  |  |  | -.03 | -.03 | .01 |
| CON-l amygdala FC |  |  |  |  | .55 | .01 |
| CON-r amygdala FC |  |  |  |  |  | .03 |

Study 1 Note. All measures are residualized for data collection site. Gray matter measures were further residualized for total brain volume. ACC = anterior cingulate cortex; CT = cortical thickness; CA = cortical area; FA = fractional anisotropy; SC = subcortical volume CON = cingulo-opercular network; l = left; r = right; FC = functional connectivity. ^$^Age correlations were corrected for sex

## Table S5. *Total, direct, and indirect family environment on amygdala-mPFC structure and function of stratified analyses in Study 1*

|  | Girls |  |  |  |  |  |  |  |  |
| --- | --- | --- | --- | --- | --- | --- | --- | --- | --- |
|  | Total effect | |  | Direct effect | |  | Indirect effect | |  |
|  | Std. Coeff. | *p* | *p_corr_* | Std. Coeff. | *p* | *p_corr_* | Std. Coeff. | *p* | *p_corr_* |
| Amygdala volume | 0.006 | .775 | .891 | 0.003 | .884 | .884 | 0.003 | .675 | .675 |
| ACC cortical thickness | -0.012 | .584 | .878 | -0.022 | .330 | .696 | 0.010 | .153 | .230 |
| ACC cortical area | 0.062 | .004 | .012 | 0.060 | .008 | .024 | 0.002 | .820 | .947 |
| L amygdala-CON | 0.108 | <.001 | <.001 | 0.090 | <.001 | <.001 | 0.018 | .014 | .028 |
| R amygdala-CON | 0.061 | .006 | .018 | 0.048 | .037 | .111 | 0.013 | .060 | .090 |
| ACC FA | -0.098 | <.001 | <.001 | -0.097 | <.001 | <.001 | 0.000 | .992 | .992 |
|  | Boys |  |  |  |  |  |  |  |  |
|  | Total effect | |  | Direct effect | |  | Indirect effect | |  |
|  | Std. Coeff. | *p* | *p_corr_* | Std. Coeff. | *p* | *p_corr_* | Std. Coeff. | *p* | *p_corr_* |
| Amygdala volume | 0.004 | .844 | .844 | 0.006 | .797 | .797 | -0.002 | .802 | .898 |
| ACC cortical thickness | -0.020 | .368 | .494 | -0.027 | .239 | .565 | 0.007 | .274 | .621 |
| ACC cortical area | 0.000 | .983 | .983 | 0.011 | .638 | .802 | -0.011 | .082 | .246 |
| L amygdala-CON | 0.092 | <.001 | <.001 | 0.088 | <.001 | <.001 | 0.004 | .559 | .570 |
| R amygdala-CON | 0.071 | .002 | .004 | .067 | .006 | .012 | 0.004 | .570 | .570 |
| ACC FA | -0.081 | .001 | .001 | -0.081 | .002 | .002 | 0.000 | .976 | .976 |

Note. ACC = anterior cingulate cortex; L = left; R = right; CON = cingulo-opercular network; FA = fractional anisotropy

|  | ACC cortical thickness | | | | ACC cortical area | | | | Amygdala volume | | | |
| --- | --- | --- | --- | --- | --- | --- | --- | --- | --- | --- | --- | --- |
|  | β | *S.E.* | β/*S.E*. | *p* | β | *S.E.* | β /*S.E.* | *p* | β | *S.E.* | β /*S.E.* | *p* |
| Fam Env^∞^ | -0.023 | 0.017 | -1.391 | .164 | 0.042 | 0.016 | 2.668 | .008 | -0.005 | 0.016 | -0.310 | .757 |
| Pubertal^+^ stage | -0.048 | 0.024 | -2.040 | .041 | 0.023 | 0.023 | 1.037 | .300 | 0.000 | 0.024 | 0.008 | .993 |
| Age | -0.105 | 0.016 | -6.585 | <.001 | 0.019 | 0.016 | 1.203 | .229 | 0.042 | 0.016 | 2.648 | .008 |
| Sex | -0.067 | 0.020 | -3.360 | .001 | -0.046 | 0.020 | -2.333 | .020 | 0.079 | 0.019 | 4.151 | <.001 |
| Race | -0.014 | 0.015 | -0.967 | .334 | 0.015 | 0.016 | 0.949 | .343 | 0.027 | 0.016 | 1.653 | .098 |
|  | Outcome: pubertal stage | | | | Outcome: pubertal stage | | | | Outcome: pubertal stage | | | |
| Fam Env^ƒ^ | -0.206 | 0.015 | -14.053 | <.001 | -0.206 | 0.015 | -14.053 | <.001 | -0.206 | 0.015 | -14.053 | <.001 |
| Age | 0.214 | 0.015 | 14.481 | <.001 | 0.214 | 0.015 | 14.481 | <.001 | 0.214 | 0.015 | 14.481 | <.001 |
| Sex | -0.523 | 0.012 | -45.056 | <.001 | -0.523 | 0.012 | -45.056 | <.001 | -0.523 | 0.012 | -45.056 | <.001 |
| Race | 0.096 | 0.015 | 6.540 | <.001 | 0.096 | 0.015 | 6.540 | <.001 | 0.096 | 0.015 | 6.540 | <.001 |

## Table S6. *Mediation model parameters – anterior cingulate cortical thickness and area, and amygdala volume Study 1*

Note. ACC = anterior cingulate cortex; ^∞^ = direct effect; ^+^ = indirect effect step 2; ^ƒ^ = indirect effect step 1.

## Table S7. *Mediation model parameters – anterior cingulate cortical thickness and surface area and amygdala volume Study 1 in girls*

|  | ACC cortical thickness | | | | ACC cortical area | | | | Amygdala volume | | | |
| --- | --- | --- | --- | --- | --- | --- | --- | --- | --- | --- | --- | --- |
|  | β | *S.E.* | β/*S.E*. | *p* | β | *S.E.* | β /*S.E.* | *p* | Β | *S.E.* | β /*S.E.* | *p* |
| Fam Env^∞^ | -0.022 | 0.023 | -0.974 | .330 | 0.060 | 0.022 | 2.672 | .008 | 0.003 | 0.023 | 0.146 | .884 |
| Pubertal stage^+^ | -0.042 | 0.029 | -1.469 | .142 | -0.007 | 0.029 | -0.231 | .817 | -0.012 | 0.028 | -0.424 | .671 |
| Age | -0.122 | 0.024 | -5.110 | <.001 | 0.040 | 0.023 | 1.716 | .086 | 0.053 | 0.023 | 2.263 | .024 |
| Race | -0.015 | 0.022 | -0.666 | .505 | 0.045 | 0.023 | 1.976 | .048 | 0.012 | 0.022 | 0.555 | .579 |
|  | Outcome: pubertal stage | | | | Outcome: pubertal stage | | | | Outcome: pubertal stage | | | |
| Fam Env^ƒ^ | -0.247 | 0.024 | -10.507 | <.001 | -0.247 | 0.024 | -10.507 | <.001 | -0.247 | 0.024 | -10.507 | <.001 |
| Age | 0.349 | 0.021 | 16.576 | <.001 | 0.349 | 0.021 | 16.576 | <.001 | 0.349 | 0.021 | 16.576 | <.001 |
| Race | 0.095 | 0.024 | 3.917 | <.001 | 0.095 | 0.024 | 3.917 | <.001 | 0.095 | 0.024 | 3.917 | <.001 |

Note. ^∞^ = direct effect; ^+^ = indirect effect step 2; ^ƒ^ = indirect effect step 1; ACC = anterior cingulate cortex

## Table S8. *Mediation model parameters – anterior cingulate cortical thickness and surface area and amygdala volume Study 1 in boys*

|  | ACC cortical thickness | | | | ACC cortical area | | | | Amygdala volume | | | |
| --- | --- | --- | --- | --- | --- | --- | --- | --- | --- | --- | --- | --- |
|  | β | *S.E.* | β/*S.E*. | *p* | β | *S.E.* | β /*S.E.* | *p* | β | *S.E.* | β /*S.E.* | *p* |
| Fam Env^∞^ | -0.027 | 0.023 | -1.177 | .239 | 0.011 | 0.022 | 0.470 | .638 | 0.006 | 0.022 | 0.257 | .797 |
| Pubertal stage^+^ | -0.030 | 0.027 | -1.109 | .268 | 0.048 | 0.027 | 1.788 | .074 | 0.007 | 0.028 | 0.253 | .801 |
| Age | -0.089 | 0.022 | -4.072 | <.001 | 0.006 | 0.022 | 0.268 | .789 | 0.037 | 0.022 | 1.708 | .088 |
| Race | -0.004 | 0.021 | -0.210 | .833 | -0.007 | 0.021 | -0.348 | .728 | 0.027 | 0.022 | 1.236 | .217 |
|  | Outcome: pubertal stage | | | | Outcome: pubertal stage | | | | Outcome: pubertal stage | | | |
| Fam Env^ƒ^ | -0.229 | 0.024 | -9.715 | <.001 | -0.229 | 0.024 | -9.715 | <.001 | -0.229 | 0.024 | -9.715 | <.001 |
| Age | 0.124 | 0.025 | 5.027 | <.001 | 0.124 | 0.025 | 5.027 | <.001 | 0.124 | 0.025 | 5.027 | <.001 |
| Race | 0.124 | 0.024 | 5.225 | <.001 | 0.124 | 0.024 | 5.225 | <.001 | 0.124 | 0.024 | 5.225 | <.001 |

Note. ^∞^ = direct effect; ^+^ = indirect effect step 2; ^ƒ^ = indirect effect step 1; ACC = anterior cingulate cortex

## Table S9. *Mediation model parameters – cinculo-opercular network-amygdala connectivity*

|  | CON-left amygdala | | | | CON-right amygdala | | | |
| --- | --- | --- | --- | --- | --- | --- | --- | --- |
|  | β | *S.E.* | β/*S.E*. | *p* | β | *S.E.* | β /*S.E.* | *p* |
| Family Environment^∞^ | 0.089 | 0.017 | 5.298 | <.001 | 0.057 | 0.017 | 3.340 | .001 |
| Pubertal stage^+^ | -0.062 | 0.024 | -2.551 | .011 | -0.050 | 0.025 | -1.965 | .049 |
| Age | 0.018 | 0.016 | 1.109 | .268 | 0.022 | 0.016 | 1.345 | .179 |
| Sex | 0.005 | 0.020 | 0.255 | .799 | -0.022 | 0.020 | -1.098 | .272 |
| Race | -0.017 | 0.016 | -1.061 | .288 | -0.001 | 0.015 | -0.044 | .965 |
|  | Outcome: pubertal stage | | | | Outcome: pubertal stage | | | |
| Family Environment^ƒ^ | -0.192 | 0.016 | -12.192 | <.001 | -0.192 | 0.016 | -12.192 | <.001 |
| Age | 0.218 | 0.015 | 14.488 | <.001 | 0.218 | 0.015 | 14.488 | <.001 |
| Sex | -0.526 | 0.012 | -45.327 | <.001 | -0.526 | 0.012 | -45.327 | <.001 |
| Race | 0.090 | 0.015 | 5.946 | <.001 | 0.090 | 0.015 | 5.946 | <.001 |

Note. CON = cingulo-opercular network; ^∞^ = direct effect; ^+^ = indirect effect step 2; ^ƒ^ = indirect effect step 1.

## Table S10*. Mediation model parameters – cinculo-opercular network-amygdala connectivity in girls*

|  | CON-left amygdala | | | | CON-right amygdala | | | |
| --- | --- | --- | --- | --- | --- | --- | --- | --- |
|  | β | *S.E.* | β/*S.E*. | *p* | β | *S.E.* | β /*S.E.* | *p* |
| Family Environment^∞^ | 0.090 | 0.023 | 3.907 | <.001 | 0.048 | 0.023 | 2.086 | .037 |
| Pubertal stage^+^ | -0.076 | 0.030 | -2.566 | .010 | -0.055 | 0.029 | -1.929 | .054 |
| Age | 0.003 | 0.023 | 0.143 | .887 | -0.019 | 0.024 | -0.802 | .423 |
| Race | 0.002 | 0.022 | 0.101 | .920 | 0.009 | 0.023 | 0.394 | .694 |
|  | Outcome: pubertal stage | | | | Outcome: pubertal stage | | | |
| Family Environment^ƒ^ | -0.239 | 0.024 | -10.026 | <.001 | -0.239 | 0.024 | -10.026 | <.001 |
| Age | 0.358 | 0.022 | 16.497 | <.001 | 0.358 | 0.022 | 16.497 | <.001 |
| Race | 0.101 | 0.025 | 4.056 | <.001 | 0.101 | 0.025 | 4.056 | <.001 |

Note. ^∞^ = direct effect; ^+^ = indirect effect step 2; ^ƒ^ = indirect effect step 1; CON = cingulo-opercular network

## Table S11. *Mediation model parameters – cinculo-opercular network-amygdala connectivity in boys*

|  | CON-left amygdala | | | | CON-right amygdala | | | |
| --- | --- | --- | --- | --- | --- | --- | --- | --- |
|  | β | *S.E.* | β/*S.E*. | *p* | β | *S.E.* | β /*S.E.* | *p* |
| Family Environment^∞^ | 0.088 | 0.023 | 3.757 | <.001 | 0.067 | 0.025 | 2.729 | .006 |
| Pubertal stage^+^ | -0.018 | 0.030 | -0.587 | .557 | -0.019 | 0.033 | -0.578 | .563 |
| Age | 0.036 | 0.022 | 1.632 | .103 | 0.061 | 0.023 | 2.674 | .007 |
| Race | -0.033 | 0.024 | -1.393 | .163 | -0.012 | 0.022 | -0.522 | .602 |
|  | Outcome: pubertal stage | | | | Outcome: pubertal stage | | | |
| Family Environment^ƒ^ | -0.213 | 0.026 | -8.236 | <.001 | -0.213 | 0.026 | -8.236 | <.001 |
| Age | 0.113 | 0.027 | 4.178 | <.001 | 0.113 | 0.027 | 4.178 | <.001 |
| Race | 0.108 | 0.026 | 4.214 | <.001 | 0.108 | 0.026 | 4.214 | <.001 |

Note. ^∞^ = direct effect; ^+^ = indirect effect step 2; ^ƒ^ = indirect effect step 1; CON = cingulo-opercular network

## Table S12. *Mediation model parameters – anterior cingulate white matter fractional anisotropy*

|  | Β | *S.E.* | β/*S.E*. | *p* |
| --- | --- | --- | --- | --- |
| Family Environment^∞^ | -0.091 | 0.018 | -5.065 | <.001 |
| Pubertal stage^+^ | 0.000 | 0.026 | 0.002 | .998 |
| Age | 0.017 | 0.018 | 0.960 | .337 |
| Sex | -0.048 | 0.022 | -2.174 | .030 |
| Race | 0.042 | 0.017 | 2.427 | .015 |
|  |  | | | |
| Family Environment^ƒ^ | -0.199 | 0.017 | -11.962 | <.001 |
| Age | 0.214 | 0.017 | 12.809 | <.001 |
| Sex | -0.524 | 0.013 | -41.279 | <.001 |
| Race | 0.108 | 0.017 | 6.401 | <.001 |

Note. ^∞^ = direct effect; ^+^ = indirect effect step 2; ^ƒ^ = indirect effect step 1.

## Table S13. *Mediation model parameters – anterior cingulate white matter fractional anisotropy in girls*

|  | β | *S.E.* | β/*S.E*. | *p* |
| --- | --- | --- | --- | --- |
| Family Environment^∞^ | -0.097 | 0.026 | -3.735 | <.001 |
| Pubertal stage^+^ | 0.000 | 0.032 | 0.011 | 0.991 |
| Age | -0.008 | 0.027 | -0.300 | 0.764 |
| Race | 0.032 | 0.026 | 1.242 | 0.214 |
|  | Outcome: pubertal stage | | | |
| Family Environment^ƒ^ | -0.232 | 0.027 | -8.537 | <.001 |
| Age | 0.334 | 0.024 | 14.081 | <.001 |
| Race | 0.115 | 0.027 | 4.301 | <.001 |

Note. ^∞^ = direct effect; ^+^ = indirect effect step 2; ^ƒ^ = indirect effect step 1.

## Table S14. *Mediation model parameters – anterior cingulate white matter fractional anisotropy in boys*

|  | β | *S.E.* | β/*S.E*. | *p* |
| --- | --- | --- | --- | --- |
| Family Environment^∞^ | -0.081 | 0.026 | -3.143 | .002 |
| Pubertal stage^+^ | 0.001 | 0.032 | 0.030 | .976 |
| Age | 0.023 | 0.024 | 0.937 | .349 |
| Race | 0.051 | 0.024 | 2.126 | .034 |
|  | Outcome: pubertal stage | | | |
| Family Environment^ƒ^ | -0.239 | 0.028 | -8.423 | <.001 |
| Age | 0.140 | 0.030 | 4.611 | <.001 |
| Race | 0.137 | 0.029 | 4.674 | <.001 |

Note. ^∞^ = direct effect; ^+^ = indirect effect step 2; ^ƒ^ = indirect effect step 1.

# **Results Study 2. Wave 2 analyses**

## Table S15. *Sex-corrected correlations between outcome-variables wave 2*

|  | ACC CA | Amygdala SV | CON-l amygdala FC | CON-r amygdala FC | Age wave 1^$^ |
| --- | --- | --- | --- | --- | --- |
| ACC CT | -.08 | .07 | .01 | .01 | -.14 |
| ACC CA |  | -.06 | -.04 | -.02 | .03 |
| Amygdala SV |  |  | -.08 | -.09 | .02 |
| CON-l amygdala FC |  |  |  | .65 | -.06 |
| CON-r amygdala FC |  |  |  |  | -.03 |

Note. All measures are residualized for data collection site. Gray matter measures were further residualized for total brain volume. ACC = anterior cingulate cortex; CT = cortical thickness; CA = cortical area; FA = fractional anisotropy; SC = subcortical volume CON = cingulo-opercular network; l = left; r = right; FC = functional connectivity. ^$^Age correlations were corrected for sex

## Table S16. *Total, direct, and indirect family environment on amygdala-mPFC structure and function of stratified analyses in wave 2*

|  | Girls |  |  |  |  |  |  |  |  |
| --- | --- | --- | --- | --- | --- | --- | --- | --- | --- |
|  | Total effect | |  | Direct effect | |  | Indirect effect | |  |
|  | Std. Coeff. | *p* | *p_corr_* | Std. Coeff. | *p* | *p_corr_* | Std. Coeff. | *p* | *p_corr_* |
| Amygdala volume | -0.010 | .741 | .891 | -0.017 | .622 | .884 | 0.006 | .530 | .675 |
| ACC cortical thickness | -0.005 | .878 | .878 | -0.025 | .464 | .696 | 0.020 | .046 | .138 |
| ACC cortical area | 0.022 | .548 | .670 | 0.022 | .540 | .540 | -0.015 | .947 | .947 |
| L amygdala-CON | 0.048 | .168 | .336 | 0.025 | .478 | .894 | 0.023 | .014 | .028 |
| R amygdala-CON | 0.031 | .353 | .424 | 0.018 | .602 | .894 | 0.013 | .124 | .149 |
|  | Boys |  |  |  |  |  |  |  |  |
|  | Total effect | |  | Direct effect | |  | Indirect effect | |  |
|  | Std. Coeff. | *p* | *p_corr_* | Std. Coeff. | *p* | *p_corr_* | Std. Coeff. | *p* | *p_corr_* |
| Amygdala volume | 0.067 | .030 | .090 | .045 | .045 | .135 | .005 | .436 | .898 |
| ACC cortical thickness | 0.021 | .494 | .494 | 0.018 | .565 | .565 | 0.003 | .621 | .621 |
| ACC cortical area | -0.014 | .668 | .983 | -0.008 | .802 | .802 | -0.005 | .365 | .548 |
| L amygdala-CON | 0.096 | .001 | .003 | 0.087 | .004 | .012 | 0.009 | .131 | .347 |
| R amygdala-CON | 0.066 | .026 | .026 | 0.058 | .052 | .052 | 0.008 | .214 | .347 |

Note. ACC = anterior cingulate cortex; L = left; R = right; CON = cingulo-opercular network.

## Table S17. *Mediation model parameters – anterior cingulate cortical thickness and area, and amygdala volume wave 2*

|  | ACC cortical thickness | | | | ACC cortical area | | | | Amygdala volume | | | |
| --- | --- | --- | --- | --- | --- | --- | --- | --- | --- | --- | --- | --- |
|  | β | *S.E.* | β/*S.E*. | *p* | β | *S.E.* | β /*S.E.* | *p* | β | *S.E.* | β /*S.E.* | *p* |
| Fam Env^∞^ | -0.003 | 0.023 | -0.114 | .909 | 0.006 | 0.024 | 0.256 | .798 | 0.028 | 0.023 | 1.186 | .236 |
| Pubertal^+^ stage | -0.066 | 0.035 | -1.859 | .063 | 0.023 | 0.035 | 0.671 | .502 | -0.029 | 0.035 | -0.837 | .403 |
| Age | -0.107 | 0.024 | -4.488 | <.001 | 0.013 | 0.025 | 0.520 | .603 | 0.027 | 0.024 | 1.090 | .276 |
| Sex | -0.039 | 0.029 | -1.308 | .191 | -0.080 | 0.029 | -2.790 | .005 | 0.098 | 0.029 | 3.331 | .001 |
| Race | -0.037 | 0.024 | -1.547 | .122 | 0.024 | 0.023 | 1.075 | .282 | 0.042 | 0.024 | 1.727 | .084 |
|  | Outcome: pubertal stage | | | | Outcome: pubertal stage | | | | Outcome: pubertal stage | | | |
| Fam Env^ƒ^ | -0.161 | 0.023 | -7.122 | <.001 | -0.161 | 0.023 | -7.122 | <.001 | -0.161 | 0.023 | -7.122 | <.001 |
| Age | 0.241 | 0.024 | 10.124 | <.001 | 0.241 | 0.024 | 10.124 | <.001 | 0.241 | 0.024 | 10.124 | <.001 |
| Sex | -0.492 | 0.018 | -27.469 | <.001 | -0.492 | 0.018 | -27.469 | <.001 | -0.492 | 0.018 | -27.469 | <.001 |
| Race | 0.150 | 0.022 | 6.795 | <.001 | 0.150 | 0.022 | 6.795 | <.001 | 0.150 | 0.022 | 6.795 | <.001 |

Note. ACC = anterior cingulate cortex; ^∞^ = direct effect; ^+^ = indirect effect step 2; ^ƒ^ = indirect effect step 1.

## Table S18. *Mediation model parameters – anterior cingulate cortical thickness and surface area and amygdala volume in girls*

|  | ACC cortical thickness | | | | ACC cortical area | | | | Amygdala volume | | | |
| --- | --- | --- | --- | --- | --- | --- | --- | --- | --- | --- | --- | --- |
|  | β | *S.E.* | β/*S.E*. | *p* | β | *S.E.* | β /*S.E.* | *p* | β | *S.E.* | β /*S.E.* | *p* |
| Fam Env^∞^ | -0.025 | 0.035 | -0.733 | .464 | 0.022 | 0.036 | 0.613 | 0.540 | -0.017 | 0.034 | -0.493 | 0.622 |
| Pubertal stage^+^ | -0.091 | 0.043 | -2.125 | .034 | 0.003 | 0.046 | 0.068 | 0.946 | -0.028 | 0.043 | -0.645 | 0.519 |
| Age | -0.072 | 0.035 | -2.078 | .038 | 0.008 | 0.039 | 0.211 | 0.833 | 0.060 | 0.035 | 1.736 | 0.083 |
| Race | -0.005 | 0.033 | -0.160 | .873 | -0.007 | 0.034 | -0.216 | 0.829 | 0.022 | 0.034 | 0.648 | 0.517 |
|  | Outcome: pubertal stage | | | | Outcome: pubertal stage | | | | Outcome: pubertal stage | | | |
| Fam Env^ƒ^ | -0.224 | 0.035 | -6.448 | <.001 | -0.224 | 0.035 | -6.448 | <.001 | -0.224 | 0.035 | -6.448 | <.001 |
| Age | 0.319 | 0.033 | 9.607 | <.001 | 0.319 | 0.033 | 9.607 | <.001 | 0.319 | 0.033 | 9.607 | <.001 |
| Race | 0.138 | 0.034 | 4.016 | <.001 | 0.138 | 0.034 | 4.016 | <.001 | 0.138 | 0.034 | 4.016 | <.001 |

Note. ^∞^ = direct effect; ^+^ = indirect effect step 2; ^ƒ^ = indirect effect step 1; ACC = anterior cingulate cortex

## Table S19. *Mediation model parameters – anterior cingulate cortical thickness and surface area and amygdala volume in boys*

|  | ACC cortical thickness | | | | ACC cortical area | | | | Amygdala volume | | | |
| --- | --- | --- | --- | --- | --- | --- | --- | --- | --- | --- | --- | --- |
|  | β | *S.E.* | β/*S.E*. | *p* | β | *S.E.* | β /*S.E.* | *p* | β | *S.E.* | β /*S.E.* | *p* |
| Fam Env^∞^ | 0.018 | 0.031 | 0.575 | .565 | -0.008 | 0.033 | -0.251 | .802 | 0.062 | 0.031 | 2.003 | .045 |
| Pubertal stage^+^ | -0.022 | 0.043 | -0.518 | .605 | 0.042 | 0.041 | 1.028 | .304 | -0.037 | 0.043 | -0.863 | .388 |
| Age | -0.140 | 0.033 | -4.265 | <.001 | 0.021 | 0.034 | 0.612 | .541 | 0.002 | 0.033 | 0.066 | .947 |
| Race | -0.070 | 0.032 | -2.166 | .030 | 0.050 | 0.032 | 1.554 | .120 | 0.060 | 0.034 | 1.787 | .074 |
|  | Outcome: pubertal stage | | | | Outcome: pubertal stage | | | | Outcome: pubertal stage | | | |
| Fam Env^ƒ^ | -0.129 | 0.042 | -3.079 | .002 | -0.129 | 0.042 | -3.079 | .002 | -0.129 | 0.042 | -3.079 | .002 |
| Age | 0.214 | 0.038 | 5.703 | <.001 | 0.214 | 0.038 | 5.703 | <.001 | 0.214 | 0.038 | 5.703 | <.001 |
| Race | 0.210 | 0.034 | 6.156 | <.001 | 0.210 | 0.034 | 6.156 | <.001 | 0.210 | 0.034 | 6.156 | <.001 |

Note. ^∞^ = direct effect; ^+^ = indirect effect step 2; ^ƒ^ = indirect effect step 1; ACC = anterior cingulate cortex

## Table S20. *Mediation model parameters – cinculo-opercular network-amygdala connectivity study 2*

|  | CON-left amygdala | | | | CON-right amygdala | | | |
| --- | --- | --- | --- | --- | --- | --- | --- | --- |
|  | β | *S.E.* | β/*S.E*. | *p* | β | *S.E.* | β /*S.E.* | *p* |
| Family Environment^∞^ | 0.064 | 0.024 | 2.682 | .007 | 0.043 | 0.023 | 1.909 | .056 |
| Pubertal stage^+^ | -0.111 | 0.034 | -3.210 | .001 | -0.073 | 0.034 | -2.114 | .035 |
| Age | -0.030 | 0.023 | -1.319 | .187 | -0.015 | 0.023 | -0.664 | .507 |
| Sex | -0.040 | 0.030 | -1.332 | .183 | -0.054 | 0.028 | -1.885 | .059 |
| Race | -0.053 | 0.023 | -2.305 | .021 | -0.049 | 0.023 | -2.165 | 0.03 |
|  | Outcome: pubertal stage | | | | Outcome: pubertal stage | | | |
| Family Environment^ƒ^ | -0.137 | 0.022 | -6.343 | <.001 | -0.137 | 0.022 | -6.343 | <.001 |
| Age | 0.254 | 0.022 | 11.773 | <.001 | 0.254 | 0.022 | 11.773 | <.001 |
| Sex | -0.506 | 0.017 | -29.419 | <.001 | -0.506 | 0.017 | -29.419 | <.001 |
| Race | 0.139 | 0.023 | 6.013 | <.001 | 0.139 | 0.023 | 6.013 | <.001 |

Note. CON = cingulo-opercular network; ^∞^ = direct effect; ^+^ = indirect effect step 2; ^ƒ^ = indirect effect step 1.

## Table S21. *Mediation model parameters – cinculo-opercular network-amygdala connectivity in girls*

|  | CON-left amygdala | | | | CON-right amygdala | | | |
| --- | --- | --- | --- | --- | --- | --- | --- | --- |
|  | β | *S.E.* | β/*S.E*. | *p* | β | *S.E.* | β /*S.E.* | *p* |
| Family Environment^∞^ | 0.025 | 0.035 | 0.709 | .478 | 0.018 | 0.034 | 0.522 | .602 |
| Pubertal stage^+^ | -0.127 | 0.044 | -2.870 | .004 | -0.072 | 0.044 | -1.650 | .099 |
| Age | -0.048 | 0.035 | -1.361 | .173 | -0.008 | 0.036 | -0.228 | .820 |
| Race | -0.086 | 0.034 | -2.529 | .001 | -0.073 | 0.034 | -2.182 | .029 |
|  | Outcome: pubertal stage | | | | Outcome: pubertal stage | | | |
| Family Environment^ƒ^ | -0.183 | 0.034 | 5.315 | <.001 | -0.183 | 0.034 | 5.315 | <.001 |
| Age | 0.326 | 0.034 | 9.588 | <.001 | 0.326 | 0.034 | 9.588 | <.001 |
| Race | 0.116 | 0.035 | 3.341 | .001 | 0.116 | 0.035 | 3.341 | .001 |

Note. ^∞^ = direct effect; ^+^ = indirect effect step 2; ^ƒ^ = indirect effect step 1; CON = cingulo-opercular network

## Table S22. *Mediation model parameters – cinculo-opercular network-amygdala connectivity in boys*

|  | CON-left amygdala | | | | CON-right amygdala | | | |
| --- | --- | --- | --- | --- | --- | --- | --- | --- |
|  | β | *S.E.* | β/*S.E*. | *p* | β | *S.E.* | β /*S.E.* | *p* |
| Family Environment^∞^ | 0.087 | 0.031 | 2.861 | .004 | 0.058 | 0.030 | 1.941 | .052 |
| Pubertal stage^+^ | -0.071 | 0.042 | -1.708 | .088 | -0.061 | 0.044 | -1.384 | .166 |
| Age | -0.013 | 0.031 | -0.405 | .685 | -0.015 | 0.030 | -0.490 | .624 |
| Race | -0.027 | 0.032 | -0.836 | .403 | -0.025 | 0.033 | -0.770 | .442 |
|  | Outcome: pubertal stage | | | | Outcome: pubertal stage | | | |
| Family Environment^ƒ^ | -0.127 | 0.039 | -3.232 | .001 | -0.127 | 0.039 | -3.232 | .001 |
| Age | 0.250 | 0.036 | 6.863 | <.001 | 0.250 | 0.036 | 6.863 | <.001 |
| Race | 0.214 | 0.033 | 6.488 | <.001 | 0.214 | 0.033 | 6.488 | <.001 |

Note. ^∞^ = direct effect; ^+^ = indirect effect step 2; ^ƒ^ = indirect effect step 1; CON = cingulo-opercular network

# **Study 2. Neural change analyses**

## **Supplemental Text 4. Wave 2 analyses**

### *Structural MRI*

Amygdala Volume

When examining the associations between wave 1 family environment, wave 1 pubertal stage and amygdala development from wave 1 to wave 2, no significant associations were found, β = 0.004, p_corr_ = .868, β = 0.002, p_corr_ = .994, β = 0.001, p_corr_ = .994, for total, direct and indirect effect, respectively. For results of the sex stratified analyses, please see Table S23.

ACC Cortical Thickness

When examining the change in ACC cortical thickness data in the original sample, no significant associations were found, β = -0.004, p_corr_ = .863, β = -0.005, p_corr_ = .909, β = 0.001, p_corr_ = .794, for total, direct and indirect effect, respectively. For results of the sex stratified analyses, please see Table S23.

ACC Cortical Surface Area

For ACC cortical surface area, no significant effects were found, β = 0.011, p_corr_ = .634, β = 0.016, p_corr_ = .749, β = -0.005, p_corr_ = .819, for total, direct and indirect effect, respectively. For results of the sex stratified analyses, please see Table S23.

## Table S23. *Total, direct, and indirect family environment on amygdala-mPFC structure and function of stratified wave 2-wave 1 difference scores*

|  | Girls |  |  |  |  |  |  |  |  |
| --- | --- | --- | --- | --- | --- | --- | --- | --- | --- |
|  | Total effect | |  | Direct effect | |  | Indirect effect | |  |
|  | Std. Coeff. | *p* | *p_corr_* | Std. Coeff. | *p* | *p_corr_* | Std. Coeff. | *p* | *p_corr_* |
| Amygdala volume | -0.004 | .891 | .891 | -0.010 | .756 | .884 | 0.006 | .566 | .675 |
| ACC cortical thickness | 0.018 | .589 | .878 | 0.009 | .784 | .784 | 0.009 | .385 | .385 |
| ACC cortical area | 0.014 | .670 | .670 | 0.029 | .390 | .540 | -0.015 | .144 | .432 |
| L amygdala-CON | 0.024 | .343 | .424 | 0.008 | .745 | .894 | 0.016 | .013 | .028 |
| R amygdala-CON | 0.011 | .642 | .642 | 0.003 | .909 | .909 | 0.008 | .166 | .166 |
|  | Boys |  |  |  |  |  |  |  |  |
|  | Total effect | |  | Direct effect | |  | Indirect effect | |  |
|  | Std. Coeff. | *p* | *p_corr_* | Std. Coeff. | *p* | *p_corr_* | Std. Coeff. | *p* | *p_corr_* |
| Amygdala volume | 0.011 | .709 | .844 | .011 | .696 | .797 | -0.001 | .898 | .898 |
| ACC cortical thickness | -0.021 | .464 | .494 | -0.018 | .547 | .565 | -0.003 | .529 | .621 |
| ACC cortical area | 0.010 | .751 | .983 | 0.009 | .798 | .802 | 0.002 | .742 | .742 |
| L amygdala-CON | 0.065 | .003 | .005 | 0.059 | .008 | .012 | 0.006 | .204 | .347 |
| R amygdala-CON | 0.048 | .023 | .026 | 0.042 | .044 | .053 | 0.005 | .231 | .347 |

Note. ACC = anterior cingulate cortex; L = left; R = right; CON = cingulo-opercular network.

## Table S24. *Mediation model parameters – anterior cingulate cortical thickness and surface area and amygdala volume wave 2-wave 1 change in total sample*

|  | ACC cortical thickness | | | | ACC cortical area | | | | Amygdala volume | | | |
| --- | --- | --- | --- | --- | --- | --- | --- | --- | --- | --- | --- | --- |
|  | β | *S.E.* | β/*S.E*. | *p* | β | *S.E.* | β /*S.E.* | *p* | β | *S.E.* | β /*S.E.* | *p* |
| Fam Env^∞^ | -0.005 | 0.022 | -0.229 | .819 | 0.016 | 0.024 | 0.676 | .499 | 0.002 | 0.022 | 0.108 | .914 |
| Pubertal stage^+^ | -0.009 | 0.032 | -0.265 | .791 | 0.030 | 0.033 | 0.922 | .357 | -0.007 | 0.033 | -0.230 | .818 |
| Age | -0.052 | 0.023 | -2.261 | .024 | -0.013 | 0.024 | -0.565 | .572 | 0.019 | 0.022 | 0.868 | .386 |
| Sex | 0.048 | 0.027 | 1.787 | .074 | 0.007 | 0.027 | 0.262 | .794 | 0.034 | 0.027 | 1.229 | .219 |
| Race | -0.024 | 0.023 | -1.024 | .306 | 0.019 | 0.026 | 0.747 | .455 | 0.002 | 0.022 | 0.084 | .933 |
| ACC thickness wave 1 | -0.400 | 0.022 | -18.215 | <.001 | 0.057 | 0.022 | 2.576 | .010 |  |  |  |  |
| ACC area wave 1 | -0.031 | 0.020 | -1.522 | .128 | -0.166 | 0.023 | -7.288 | <.001 |  |  |  |  |
| Amygdala wave 1 |  |  |  |  |  |  |  |  | -0.364 | 0.025 | -14.771 | <.001 |
|  | Outcome: pubertal stage | | | | Outcome: pubertal stage | | | | Outcome: pubertal stage | | | |
| Fam Env^ƒ^ | -0.161 | 0.024 | -6.824 | <.001 | -0.161 | 0.024 | -6.824 | <.001 | -0.161 | 0.024 | -6.824 | <.001 |
| Age | 0.236 | 0.023 | 10.374 | <.001 | 0.236 | 0.023 | 10.374 | <.001 | 0.242 | 0.022 | 10.740 | <.001 |
| Sex | -0.495 | 0.018 | -27.333 | <.001 | -0.495 | 0.018 | -27.333 | <.001 | -0.489 | 0.018 | -26.846 | <.001 |
| Race | 0.148 | 0.022 | 6.863 | <.001 | 0.148 | 0.022 | 6.863 | <.001 | 0.151 | 0.022 | 7.006 | <.001 |

Note. ^∞^ = direct effect; ^+^ = indirect effect step 2; ^ƒ^ = indirect effect step 1; ACC = anterior cingulate cortex

## Table S25. *Mediation model parameters – anterior cingulate cortical thickness and surface area and amygdala volume wave 2-wave 1 change in girls*

|  | ACC cortical thickness | | | | ACC cortical area | | | | Amygdala volume | | | |
| --- | --- | --- | --- | --- | --- | --- | --- | --- | --- | --- | --- | --- |
|  | β | *S.E.* | β/*S.E*. | *p* | β | *S.E.* | β /*S.E.* | *p* | β | *S.E.* | β /*S.E.* | *p* |
| Fam Env^∞^ | 0.009 | 0.034 | 0.274 | .784 | 0.029 | 0.034 | 0.860 | .390 | 0.009 | 0.034 | 0.261 | .794 |
| Pubertal stage^+^ | -0.039 | 0.043 | -0.906 | .370 | 0.066 | 0.042 | 1.563 | .118 | -0.037 | 0.043 | -0.861 | .389 |
| Age | -0.019 | 0.035 | -0.545 | .586 | -0.010 | 0.035 | -0.287 | .774 | -0.020 | 0.035 | 0.559 | .576 |
| Race | 0.013 | 0.033 | 0.413 | .679 | -0.002 | 0.035 | -0.068 | .946 | 0.014 | 0.033 | 0.422 | .673 |
| ACC thickness wave 1 | -0.385 | 0.029 | -13.409 | <.001 | 0.080 | 0.029 | 2.782 | .005 |  |  |  |  |
| ACC area wave 1 | -0.048 | 0.030 | -1.610 | .107 | -0.153 | 0.034 | -4.475 | <.001 |  |  |  |  |
| Amygdala wave 1 |  |  |  |  |  |  |  |  | -0.380 | 0.029 | 13.280 | <.001 |
|  | Outcome: pubertal stage | | | | Outcome: pubertal stage | | | | Outcome: pubertal stage | | | |
| Fam Env^ƒ^ | -0.225 | 0.034 | -6.576 | <.001 | -0.225 | 0.034 | -6.576 | <.001 | -0.225 | 0.034 | -6.577 | <.001 |
| Age | 0.314 | 0.033 | 9.535 | <.001 | 0.314 | 0.033 | 9.535 | <.001 | 0.313 | 0.033 | 9.533 | <.001 |
| Race | 0.137 | 0.036 | 3.811 | <.001 | 0.137 | 0.036 | 3.811 | <.001 | 0.137 | 0.036 | 3.831 | <.001 |

Note. ^∞^ = direct effect; ^+^ = indirect effect step 2; ^ƒ^ = indirect effect step 1; ACC = anterior cingulate cortex

## Table S26. *Mediation model parameters – anterior cingulate cortical thickness and surface area and amygdala volume wave 2-wave 1 change in boys*

|  | ACC cortical thickness | | | | ACC cortical area | | | | Amygdala volume | | | |
| --- | --- | --- | --- | --- | --- | --- | --- | --- | --- | --- | --- | --- |
|  | β | *S.E.* | β/*S.E*. | *p* | β | *S.E.* | β /*S.E.* | *p* | β | *S.E.* | β /*S.E.* | *p* |
| Fam Env^∞^ | -0.018 | 0.030 | -0.602 | .547 | 0.009 | 0.033 | 0.256 | .798 | 0.011 | 0.029 | 0.391 | .696 |
| Pubertal stage^+^ | 0.027 | 0.038 | 0.692 | .489 | -0.014 | 0.041 | -0.349 | .727 | 0.005 | 0.040 | 0.135 | .893 |
| Age | -0.081 | 0.030 | -2.725 | .006 | -0.015 | 0.034 | -0.455 | .649 | -0.002 | 0.032 | -0.068 | .946 |
| Race | -0.064 | 0.031 | -2.067 | .039 | 0.041 | 0.036 | 1.147 | .251 | -0.015 | 0.031 | -0.474 | .636 |
| ACC thickness wave 1 | -0.416 | 0.031 | -13.323 | <.001 | 0.039 | 0.030 | 1.298 | .194 |  |  |  |  |
| ACC area wave 1 | -0.015 | 0.027 | -0.535 | .593 | -0.174 | 0.031 | -5.615 | <.001 |  |  |  |  |
| Amygdala wave 1 |  |  |  |  |  |  |  |  | -0.367 | 0.033 | -11.157 | <.001 |
|  | Outcome: pubertal stage | | | | Outcome: pubertal stage | | | | Outcome: pubertal stage | | | |
| Fam Env^ƒ^ | -0.126 | 0.042 | -2.979 | .003 | -0.126 | 0.042 | -2.979 | .003 | -0.125 | 0.042 | -2.972 | .003 |
| Age | 0.208 | 0.038 | 5.444 | <.001 | 0.208 | 0.038 | 5.444 | <.001 | 0.214 | 0.037 | 5.708 | <.001 |
| Race | 0.207 | 0.035 | 5.923 | <.001 | 0.207 | 0.035 | 5.923 | <.001 | 0.214 | 0.034 | 6.238 | <.001 |

Note. ^∞^ = direct effect; ^+^ = indirect effect step 2; ^ƒ^ = indirect effect step 1; ACC = anterior cingulate cortex

## Table S27. *Mediation model parameters – cinculo-opercular network-amygdala connectivity wave 2-wave 1 change in total sample*

|  | CON-left amygdala | | | | CON-right amygdala | | | |
| --- | --- | --- | --- | --- | --- | --- | --- | --- |
|  | β | *S.E.* | β/*S.E*. | *p* | β | *S.E.* | β /*S.E.* | *p* |
| Family Environment^∞^ | 0.038 | 0.017 | 2.204 | .028 | 0.025 | 0.016 | 1.529 | .126 |
| Pubertal stage^+^ | -0.078 | 0.025 | -3.024 | .002 | -0.052 | 0.025 | -2.042 | .041 |
| Age | -0.022 | 0.017 | -1.316 | .188 | -0.009 | 0.016 | -0.573 | .566 |
| Sex | -0.030 | 0.021 | -1.461 | .144 | -0.040 | 0.020 | -1.979 | .048 |
| Race | -0.038 | 0.017 | -2.208 | .027 | -0.033 | 0.017 | -1.946 | .052 |
| CON-l amygdala wave 1 | -0.679 | 0.022 | -30.837 | <.001 | 0.037 | 0.024 | 1.505 | .132 |
| CON-r amygdala wave 1 | -0.009 | 0.022 | 0.407 | .685 | -0.723 | 0.020 | 36.779 | <.001 |
|  | Outcome: pubertal stage | | | | Outcome: pubertal stage | | | |
| Family Environment^ƒ^ | -0.130 | 0.023 | -5.755 | <.001 | -0.130 | 0.023 | -5.755 | <.001 |
| Age | 0.254 | 0.022 | 11.755 | <.001 | 0.254 | 0.022 | 11.755 | <.001 |
| Sex | -0.508 | 0.018 | -28.183 | <.001 | -0.508 | 0.018 | -28.183 | <.001 |
| Race | 0.139 | 0.022 | 6.263 | <.001 | 0.139 | 0.022 | 6.263 | <.001 |

Note. CON = cingulo-opercular network; ^∞^ = direct effect; ^+^ = indirect effect step 2; ^ƒ^ = indirect effect step 1.

## Table S28. *Mediation model parameters – cinculo-opercular network-amygdala connectivity wave 2-wave 1 change in girls*

|  | CON-left amygdala | | | | CON-right amygdala | | | |
| --- | --- | --- | --- | --- | --- | --- | --- | --- |
|  | β | *S.E.* | β/*S.E*. | *p* | β | *S.E.* | β /*S.E.* | *p* |
| Family Environment^∞^ | 0.008 | 0.026 | 0.325 | .745 | 0.003 | 0.024 | 0.114 | .909 |
| Pubertal stage^+^ | -0.089 | 0.031 | -2.896 | .004 | -0.047 | 0.031 | -1.501 | .133 |
| Age | -0.035 | 0.026 | -1.356 | .175 | -0.007 | 0.024 | -0.287 | .774 |
| Race | -0.051 | 0.026 | -1.928 | .054 | -0.038 | 0.024 | -1.574 | .116 |
| CON-l amygdala wave 1 | -0.678 | 0.030 | -22.364 | <.001 | 0.011 | 0.035 | 0.327 | .744 |
| CON-r amygdala wave 1 | 0.009 | 0.030 | 0.287 | .774 | -0.719 | 0.028 | -25.595 | <.001 |
|  | Outcome: pubertal stage | | | | Outcome: pubertal stage | | | |
| Family Environment^ƒ^ | -0.175 | 0.035 | -5.076 | <.001 | -0.175 | 0.035 | -5.076 | <.001 |
| Age | 0.324 | 0.034 | 9.540 | <.001 | 0.324 | 0.034 | 9.540 | <.001 |
| Race | 0.116 | 0.037 | 3.154 | .002 | 0.116 | 0.037 | 3.154 | .002 |

Note. CON = cingulo-opercular network; ^∞^ = direct effect; ^+^ = indirect effect step 2; ^ƒ^ = indirect effect step 1.

## Table S29. *Mediation model parameters – cinculo-opercular network-amygdala connectivity wave 2-wave 1 change in boys*

|  | CON-left amygdala | | | | CON-right amygdala | | | |
| --- | --- | --- | --- | --- | --- | --- | --- | --- |
|  | β | *S.E.* | β/*S.E*. | *p* | β | *S.E.* | β /*S.E.* | *p* |
| Family Environment^∞^ | 0.059 | 0.022 | 2.667 | .008 | 0.042 | 0.021 | 2.013 | .044 |
| Pubertal stage^+^ | -0.046 | 0.031 | -1.511 | .131 | -0.044 | 0.031 | -1.415 | .157 |
| Age | -0.013 | 0.023 | 0.587 | .557 | -0.014 | 0.022 | -0.608 | .543 |
| Race | -0.028 | 0.023 | -1.218 | .223 | -0.029 | 0.024 | -1.199 | .231 |
| CON-l amygdala wave 1 | -0.681 | 0.030 | -23.055 | <.001 | 0.057 | 0.033 | 1.729 | .084 |
| CON-r amygdala wave 1 | 0.010 | 0.029 | 0.354 | .723 | -0.728 | 0.030 | -24.634 | <.001 |
|  | Outcome: pubertal stage | | | | Outcome: pubertal stage | | | |
| Family Environment^ƒ^ | -0.121 | 0.038 | -3.175 | .002 | -0.121 | 0.038 | -3.175 | .002 |
| Age | 0.253 | 0.036 | 7.052 | <.001 | 0.253 | 0.036 | 7.052 | <.001 |
| Race | 0.215 | 0.034 | 6.317 | <.001 | 0.215 | 0.034 | 6.317 | <.001 |

Note. CON = cingulo-opercular network; ^∞^ = direct effect; ^+^ = indirect effect step 2; ^ƒ^ = indirect effect step 1.

## Table S30. *Split half-replication of rs-fMRI analyses in Study 2: total, direct, and indirect family environment on amygdala-mPFC functional connectivity*

|  |  | Wave 2: neurodevelopmental timing | | | | | | |
| --- | --- | --- | --- | --- | --- | --- | --- | --- |
|  |  | Total effect | | Direct effect | | Indirect effect | | |
|  |  | Std. Coeff. | *p* | Std. Coeff. | *p* | | Std. Coeff. | *p* |
| L amygdala-CON | Subsample 1 (n= 958) | 0.054 | .098 | 0.040 | .211 | | 0.014 | .045 |
| L amygdala-CON | Subsample 2  (n= 967) | 0.104 | .001 | 0.087 | .009 | | 0.017 | .063 |
| R amygdala-CON | Subsample 1 | 0.031 | .312 | 0.023 | .462 | | 0.009 | .178 |
| R amygdala-CON | Subsample 2 | 0.077 | .013 | 0.065 | .044 | | 0.012 | .185 |
|  |  | Wave 1-Wave 2 difference scores: neurodevelopmental tempo | | | | | | |
|  |  | Total effect | | Direct effect | | | Indirect effect | |
|  |  | Std. Coeff. | *p* | Std. Coeff. | *p* | | Std. Coeff. | *p* |
| L amygdala-CON | Subsample 1  (n= 958) | 0.048 | .031 | 0.035 | .117 | | 0.013 | .027 |
| L amygdala-CON | Subsample 2  (n= 954) | 0.051 | .051 | 0.042 | .111 | | 0.008 | .148 |
| R amygdala-CON | Subsample 1 | 0.009 | .661 | 0.001 | .975 | | 0.009 | .082 |
| R amygdala-CON | Subsample 2 | 0.057 | .017 | 0.051 | .033 | | 0.005 | .358 |

## Table S31. *Total, direct, and indirect family environment on amygdala-mPFC structure and function corrected for traumatic events in Study 1 and Study 2*

|  | Study 1 | | | | | | | | | |  |
| --- | --- | --- | --- | --- | --- | --- | --- | --- | --- | --- | --- |
|  | Total effect | | Direct effect | | Indirect effect | | | | | |  |
|  | Std. Coeff. | *p* | Std. Coeff. | *p* | Std. Coeff. | | *p* | | | |  |
| Amygdala volume | 0.002 | .521 | 0.003 | .432 | -0.001 | | .432 | | | |  |
| ACC cortical thickness | 0.036 | .023 | 0.040 | .015 | -0.004 | | .409 | | | |  |
| ACC cortical area | -0.004 | .793 | -0.003 | .836 | -0.001 | | .889 | | | |  |
| L amygdala-CON | 0.098 | < .001 | 0.087 | < .001 | 0.011 | | .014 | | | |  |
| R amygdala-CON | 0.066 | <.001 | 0.057 | .001 | 0.009 | | .062 | | | |  |
| ACC FA | -0.084 | < .001 | -0.084 | < .001 | 0.000 | | .973 | | | |  |
|  | Study 2 – timing effects | | | | | | | |  | | |
|  | Total effect | | Direct effect | | Indirect effect | | | | |  |  |
|  | Std. Coeff. | *p* | Std. Coeff. | *p* | Std. Coeff. | | | *p* | | | |
| Amygdala volume | 0.027 | .223 | 0.021 | .336 | 0.006 | | | .336 | | | |
| ACC cortical thickness | 0.020 | .414 | 0.011 | .667 | 0.009 | | | .152 | | | |
| ACC cortical area | 0.012 | .639 | 0.016 | .545 | -0.004 | | | .494 | | | |
| L amygdala-CON | 0.069 | .024 | 0.054 | .026 | 0.015 | | | .006 | | | |
| R amygdala-CON | 0.048 | .041 | 0.036 | .127 | 0.012 | | | .024 | | | |
|  | Study 2 – tempo effects | | | |  |  | | |  | | |
|  | Total effect |  | Direct effect | | Indirect effect | | | | | | |
|  | Std. Coeff. | *p* | Std. Coeff. | *p* | Std. Coeff. | | | *p* | | | |
| Amygdala volume | -0.002 | .926 | -0.004 | .865 | -0.004 | | .736 | | | | |
| ACC cortical thickness | 0.007 | .751 | 0.007 | .768 | 0.000 | | .940 | | | | |
| ACC cortical area | 0.017 | .467 | 0.020 | .415 | -0.003 | | .618 | | | | |
| L amygdala-CON | 0.041 | .023 | 0.031 | .099 | 0.010 | | .008 | | | | |
| R amygdala-CON | 0.029 | .081 | 0.020 | .223 | 0.008 | | .025 | | | | |

References

Achenbach, T. M., & Rescorla, L. A. (2003). *Manual for the ASEBA Adult Forms and Profiles*. University of Vermont, Research Center for Children, Youth and Families.

Barch, D. M., Albaugh, M. D., Avenevoli, S., Chang, L., Clark, D. B., Glantz, M. D., Hudziak, J. J., Jernigan, T. L., Tapert, S. F., Yurgelun-Todd, D., Alia-Klein, N., Potter, A. S., Paulus, M. P., Prouty, D., Zucker, R. A., & Sher, K. J. (2018). Demographic, physical and mental health assessments in the adolescent brain and cognitive development study: Rationale and description. *Dev Cogn Neurosci*, *32*, 55-66. <https://doi.org/10.1016/j.dcn.2017.10.010>

Basser, P. J., Mattiello, J., & LeBihan, D. (1994). Estimation of the effective self-diffusion tensor from the NMR spin echo. *J Magn Reson B*, *103*(3), 247-254. <https://doi.org/10.1006/jmrb.1994.1037>

Chilcoat, H. D., & Anthony, J. C. (1996). Impact of parent monitoring on initiation of drug use through late childhood. *J Am Acad Child Adolesc Psychiatry*, *35*(1), 91-100. <https://doi.org/10.1097/00004583-199601000-00017>

Cox, R. W. (1996). AFNI: software for analysis and visualization of functional magnetic resonance neuroimages. *Comput Biomed Res*, *29*(3), 162-173. <https://doi.org/10.1006/cbmr.1996.0014>

Dale, A. M., Fischl, B., & Sereno, M. I. (1999). Cortical surface-based analysis. I. Segmentation and surface reconstruction. *Neuroimage*, *9*(2), 179-194. <https://doi.org/10.1006/nimg.1998.0395>

Dale, A. M., & Sereno, M. I. (1993). Improved Localizadon of Cortical Activity by Combining EEG and MEG with MRI Cortical Surface Reconstruction: A Linear Approach. *J Cogn Neurosci*, *5*(2), 162-176. <https://doi.org/10.1162/jocn.1993.5.2.162>

Elman, J. A., Panizzon, M. S., Hagler, D. J., Jr., Fennema-Notestine, C., Eyler, L. T., Gillespie, N. A., Neale, M. C., Lyons, M. J., Franz, C. E., McEvoy, L. K., Dale, A. M., & Kremen, W. S. (2017). Genetic and environmental influences on cortical mean diffusivity. *Neuroimage*, *146*, 90-99. <https://doi.org/10.1016/j.neuroimage.2016.11.032>

Fischl, B., & Dale, A. M. (2000). Measuring the thickness of the human cerebral cortex from magnetic resonance images. *Proc Natl Acad Sci U S A*, *97*(20), 11050-11055. <https://doi.org/10.1073/pnas.200033797>

Fischl, B., Liu, A., & Dale, A. M. (2001). Automated manifold surgery: constructing geometrically accurate and topologically correct models of the human cerebral cortex. *IEEE Trans Med Imaging*, *20*(1), 70-80. <https://doi.org/10.1109/42.906426>

Fischl, B., Salat, D. H., Busa, E., Albert, M., Dieterich, M., Haselgrove, C., van der Kouwe, A., Killiany, R., Kennedy, D., Klaveness, S., Montillo, A., Makris, N., Rosen, B., & Dale, A. M. (2002). Whole brain segmentation: automated labeling of neuroanatomical structures in the human brain. *Neuron*, *33*(3), 341-355. <https://doi.org/10.1016/s0896-6273(02)00569-x>

Fischl, B., Sereno, M. I., Tootell, R. B., & Dale, A. M. (1999). High-resolution intersubject averaging and a coordinate system for the cortical surface. *Hum Brain Mapp*, *8*(4), 272-284. <https://doi.org/10.1002/(sici)1097-0193(1999)8:4><272::aid-hbm10>3.0.co;2-4

Gordon, E. M., Laumann, T. O., Adeyemo, B., Huckins, J. F., Kelley, W. M., & Petersen, S. E. (2016). Generation and Evaluation of a Cortical Area Parcellation from Resting-State Correlations. *Cereb Cortex*, *26*(1), 288-303. <https://doi.org/10.1093/cercor/bhu239>

Hagler, D. J., Jr., Hatton, S., Cornejo, M. D., Makowski, C., Fair, D. A., Dick, A. S., Sutherland, M. T., Casey, B. J., Barch, D. M., Harms, M. P., Watts, R., Bjork, J. M., Garavan, H. P., Hilmer, L., Pung, C. J., Sicat, C. S., Kuperman, J., Bartsch, H., Xue, F., Heitzeg, M. M., Laird, A. R., Trinh, T. T., Gonzalez, R., Tapert, S. F., Riedel, M. C., Squeglia, L. M., Hyde, L. W., Rosenberg, M. D., Earl, E. A., Howlett, K. D., Baker, F. C., Soules, M., Diaz, J., de Leon, O. R., Thompson, W. K., Neale, M. C., Herting, M., Sowell, E. R., Alvarez, R. P., Hawes, S. W., Sanchez, M., Bodurka, J., Breslin, F. J., Morris, A. S., Paulus, M. P., Simmons, W. K., Polimeni, J. R., van der Kouwe, A., Nencka, A. S., Gray, K. M., Pierpaoli, C., Matochik, J. A., Noronha, A., Aklin, W. M., Conway, K., Glantz, M., Hoffman, E., Little, R., Lopez, M., Pariyadath, V., Weiss, S. R., Wolff-Hughes, D. L., DelCarmen-Wiggins, R., Feldstein Ewing, S. W., Miranda-Dominguez, O., Nagel, B. J., Perrone, A. J., Sturgeon, D. T., Goldstone, A., Pfefferbaum, A., Pohl, K. M., Prouty, D., Uban, K., Bookheimer, S. Y., Dapretto, M., Galvan, A., Bagot, K., Giedd, J., Infante, M. A., Jacobus, J., Patrick, K., Shilling, P. D., Desikan, R., Li, Y., Sugrue, L., Banich, M. T., Friedman, N., Hewitt, J. K., Hopfer, C., Sakai, J., Tanabe, J., Cottler, L. B., Nixon, S. J., Chang, L., Cloak, C., Ernst, T., Reeves, G., Kennedy, D. N., Heeringa, S., Peltier, S., Schulenberg, J., Sripada, C., Zucker, R. A., Iacono, W. G., Luciana, M., Calabro, F. J., Clark, D. B., Lewis, D. A., Luna, B., Schirda, C., Brima, T., Foxe, J. J., Freedman, E. G., Mruzek, D. W., Mason, M. J., Huber, R., McGlade, E., Prescot, A., Renshaw, P. F., Yurgelun-Todd, D. A., Allgaier, N. A., Dumas, J. A., Ivanova, M., Potter, A., Florsheim, P., Larson, C., Lisdahl, K., Charness, M. E., Fuemmeler, B., Hettema, J. M., Maes, H. H., Steinberg, J., Anokhin, A. P., Glaser, P., Heath, A. C., Madden, P. A., Baskin-Sommers, A., Constable, R. T., Grant, S. J., Dowling, G. J., Brown, S. A., Jernigan, T. L., & Dale, A. M. (2019). Image processing and analysis methods for the Adolescent Brain Cognitive Development Study. *Neuroimage*, *202*, 116091. <https://doi.org/10.1016/j.neuroimage.2019.116091>

Hallquist, M. N., Hwang, K., & Luna, B. (2013). The nuisance of nuisance regression: spectral misspecification in a common approach to resting-state fMRI preprocessing reintroduces noise and obscures functional connectivity. *Neuroimage*, *82*, 208-225. <https://doi.org/10.1016/j.neuroimage.2013.05.116>

Holland, D., Kuperman, J. M., & Dale, A. M. (2010). Efficient correction of inhomogeneous static magnetic field-induced distortion in Echo Planar Imaging. *Neuroimage*, *50*(1), 175-183. <https://doi.org/10.1016/j.neuroimage.2009.11.044>

Jovicich, J., Czanner, S., Greve, D., Haley, E., van der Kouwe, A., Gollub, R., Kennedy, D., Schmitt, F., Brown, G., Macfall, J., Fischl, B., & Dale, A. (2006). Reliability in multi-site structural MRI studies: effects of gradient non-linearity correction on phantom and human data. *Neuroimage*, *30*(2), 436-443. <https://doi.org/10.1016/j.neuroimage.2005.09.046>

Kaufman, J., Birmaher, B., Brent, D., Rao, U., Flynn, C., Moreci, P., Williamson, D., & Ryan, N. (1997). Schedule for Affective Disorders and Schizophrenia for School-Age Children-Present and Lifetime Version (K-SADS-PL): initial reliability and validity data. *J Am Acad Child Adolesc Psychiatry*, *36*(7), 980-988. <https://doi.org/10.1097/00004583-199707000-00021>

Kessler, R. C., Avenevoli, S., Costello, E. J., Green, J. G., Gruber, M. J., Heeringa, S., Merikangas, K. R., Pennell, B. E., Sampson, N. A., & Zaslavsky, A. M. (2009). Design and field procedures in the US National Comorbidity Survey Replication Adolescent Supplement (NCS-A). *Int J Methods Psychiatr Res*, *18*(2), 69-83. <https://doi.org/10.1002/mpr.279>

Leemans, A., & Jones, D. K. (2009). The B-matrix must be rotated when correcting for subject motion in DTI data. *Magn Reson Med*, *61*(6), 1336-1349. <https://doi.org/10.1002/mrm.21890>

Moos, R. H., & Moos, B. S. (1976). A typology of family social environments. *Fam Process*, *15*(4), 357-371. <https://doi.org/10.1111/j.1545-5300.1976.00357.x>

Parkes, L., Fulcher, B., Yucel, M., & Fornito, A. (2018). An evaluation of the efficacy, reliability, and sensitivity of motion correction strategies for resting-state functional MRI. *Neuroimage*, *171*, 415-436. <https://doi.org/10.1016/j.neuroimage.2017.12.073>

Power, J. D., Barnes, K. A., Snyder, A. Z., Schlaggar, B. L., & Petersen, S. E. (2012). Spurious but systematic correlations in functional connectivity MRI networks arise from subject motion. *Neuroimage*, *59*(3), 2142-2154. <https://doi.org/10.1016/j.neuroimage.2011.10.018>

Power, J. D., Mitra, A., Laumann, T. O., Snyder, A. Z., Schlaggar, B. L., & Petersen, S. E. (2014). Methods to detect, characterize, and remove motion artifact in resting state fMRI. *Neuroimage*, *84*, 320-341. <https://doi.org/10.1016/j.neuroimage.2013.08.048>

Satterthwaite, T. D., Wolf, D. H., Loughead, J., Ruparel, K., Elliott, M. A., Hakonarson, H., Gur, R. C., & Gur, R. E. (2012). Impact of in-scanner head motion on multiple measures of functional connectivity: relevance for studies of neurodevelopment in youth. *Neuroimage*, *60*(1), 623-632. <https://doi.org/10.1016/j.neuroimage.2011.12.063>

Schaefer, E. S. (1965). Children's Reports of Parental Behavior: An Inventory. *Child Dev*, *36*, 413-424. <https://www.ncbi.nlm.nih.gov/pubmed/14300862>

Segonne, F., Dale, A. M., Busa, E., Glessner, M., Salat, D., Hahn, H. K., & Fischl, B. (2004). A hybrid approach to the skull stripping problem in MRI. *Neuroimage*, *22*(3), 1060-1075. <https://doi.org/10.1016/j.neuroimage.2004.03.032>

Segonne, F., Pacheco, J., & Fischl, B. (2007). Geometrically accurate topology-correction of cortical surfaces using nonseparating loops. *IEEE Trans Med Imaging*, *26*(4), 518-529. <https://doi.org/10.1109/TMI.2006.887364>

Seibert, T. M., & Brewer, J. B. (2011). Default network correlations analyzed on native surfaces. *J Neurosci Methods*, *198*(2), 301-311. <https://doi.org/10.1016/j.jneumeth.2011.04.010>

Stover, P. J., Harlan, W. R., Hammond, J. A., Hendershot, T., & Hamilton, C. M. (2010). PhenX: a toolkit for interdisciplinary genetics research. *Curr Opin Lipidol*, *21*(2), 136-140. <https://doi.org/10.1097/MOL.0b013e3283377395>

Thijssen, S., Collins, P. F., & Luciana, M. (2020). Pubertal development mediates the association between family environment and brain structure and function in childhood. *Dev Psychopathol*, *32*(2), 687-702. <https://doi.org/10.1017/S0954579419000580>

Van Dijk, K. R., Hedden, T., Venkataraman, A., Evans, K. C., Lazar, S. W., & Buckner, R. L. (2010). Intrinsic functional connectivity as a tool for human connectomics: theory, properties, and optimization. *J Neurophysiol*, *103*(1), 297-321. <https://doi.org/10.1152/jn.00783.2009>

Wells, W. M., 3rd, Viola, P., Atsumi, H., Nakajima, S., & Kikinis, R. (1996). Multi-modal volume registration by maximization of mutual information. *Med Image Anal*, *1*(1), 35-51. <https://doi.org/10.1016/s1361-8415(01)80004-9>

Zhuang, J., Hrabe, J., Kangarlu, A., Xu, D., Bansal, R., Branch, C. A., & Peterson, B. S. (2006). Correction of eddy-current distortions in diffusion tensor images using the known directions and strengths of diffusion gradients. *J Magn Reson Imaging*, *24*(5), 1188-1193. <https://doi.org/10.1002/jmri.20727>

Zucker, R. A., Gonzalez, R., Feldstein Ewing, S. W., Paulus, M. P., Arroyo, J., Fuligni, A., Morris, A. S., Sanchez, M., & Wills, T. (2018). Assessment of culture and environment in the Adolescent Brain and Cognitive Development Study: Rationale, description of measures, and early data. *Dev Cogn Neurosci*, *32*, 107-120. <https://doi.org/10.1016/j.dcn.2018.03.004>
